# Flexural behaviour of RC shear wall using enhanced finite element model

**DOI:** 10.1038/s41598-026-52257-5

**Published:** 2026-05-19

**Authors:** Omar Nasr, Ayman Moustafa, Ahmed H. Ghallab

**Affiliations:** Structural Engineering Department, Ain Shams University, Cairo, Egypt

**Keywords:** Reinforced concrete shear walls (RC WALLS), Finite element modeling (FEM), Experimental verification, Material modelling, Engineering, Materials science, Mathematics and computing

## Abstract

Accurate nonlinear finite element modelling (FEM) of reinforced concrete (RC) shear walls is essential for evaluating their seismic performance and ensuring structural safety. However, most existing modelling approaches often involve high computational costs or simplified representations of nonlinear behaviour such as cracking, tension stiffening, and confinement effects. This study presents a practical and verified FEM approach for RC shear walls that fail in flexure, achieving a balance between accuracy and computational efficiency. The proposed modelling strategy employs nonlinear material constitutive models for both concrete and reinforcement and utilizes a refined mesh configuration to capture the global response while maintaining manageable computation times. The analyses were performed using CSI ETABS, and the model was validated against thirteen experimental wall specimens from nine independent studies with varying geometries, reinforcement ratios, and boundary conditions. The comparison of pushover curves indicated strong agreement with experimental data. The results confirm that the proposed FEM methodology can reliably simulate the nonlinear behaviour of flexural RC shear walls.

## Introduction

Reinforced concrete (RC) shear walls are one of the main structural systems used to resist lateral loads especially in moderate to high rise buildings. Different types of failure are generally observed in cast-in-situ reinforced concrete shear walls after earthquake, such as flexural failure, shear failure (diagonal Tension failure, diagonal compression failure) and compression failure. Slender shear walls (height -to –length > 2) typically exhibited flexure-controlled failure under earthquake loads, characterized by yielding of vertical reinforcement and concrete crushing at base and significant ductile energy dissipation.

Earthquakes remain challenging due to the complexity of cracking phenomena, stiffness degradation, and the nonlinear response characteristics that must be considered in design procedures. Different methods are used to assess the response of flexural shear walls to lateral loads, including Finite Element Analysis (FEA) and analytical methods.

Pushover analysis is a static nonlinear analysis where earthquake load is considered as a static load applied at top or distributed along the structure height and increases its value gradually till failure. The main objectives of pushover analysis are determining the maximum load, maximum deformation of the structure, identifying yielding sequences, plastic hinge regions, and ultimate failure mechanisms. Pushover analysis produces capacity curve that represents the relation between base shear and top displacement.    

**Fig. 1:**
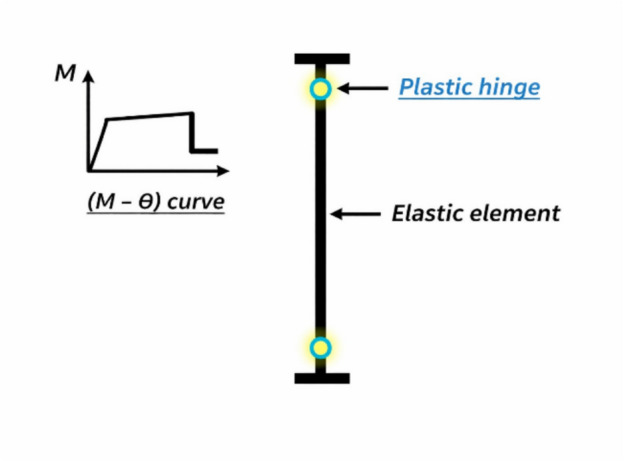
Equivalent beam–column with concentrated plastic hinge approach.

Nonlinear behavior of RC shear walls can be simulated by several modeling techniques such as^1,2^:

- *Mid-Pier (Column) element* the shear wall is modeled as a single wide column with lumped plasticity hinges at base (Fig. 1). This method is computationally efficient and suitable for tall, solid shear walls.
- *Fiber-based models* Sections are divided into discrete fibers representing concrete and steel, allowing for distributed plasticity. This approach is effective for capturing the nonlinear flexural response and material-level behavior.
- *Multi-layer shell element* a fine mesh of shell elements with distinct layers for concrete and reinforcement were used. This method captures complex in-plane bending and shear interactions more accurately.
- *Macro-models*: A combination of membrane elements for the wall panel and column elements for boundary zones to simulate barbell or flanged shapes are used

### Failure modes of RC shear walls ^3^

According to ASCE/SEI 41-17^4^, reinforced concrete (RC) shear walls are typically categorized based on their aspect ratio (h_x_/l_x_), where *h*_x_ is the wall height and *l*_x_ is the wall length. Walls with an aspect ratio greater than 3.0 are classified as slender walls, those with ratios between 1.5 and 3.0 as moderate walls, and those with ratios less than 1.5 as squat walls.

**Fig. 2:**
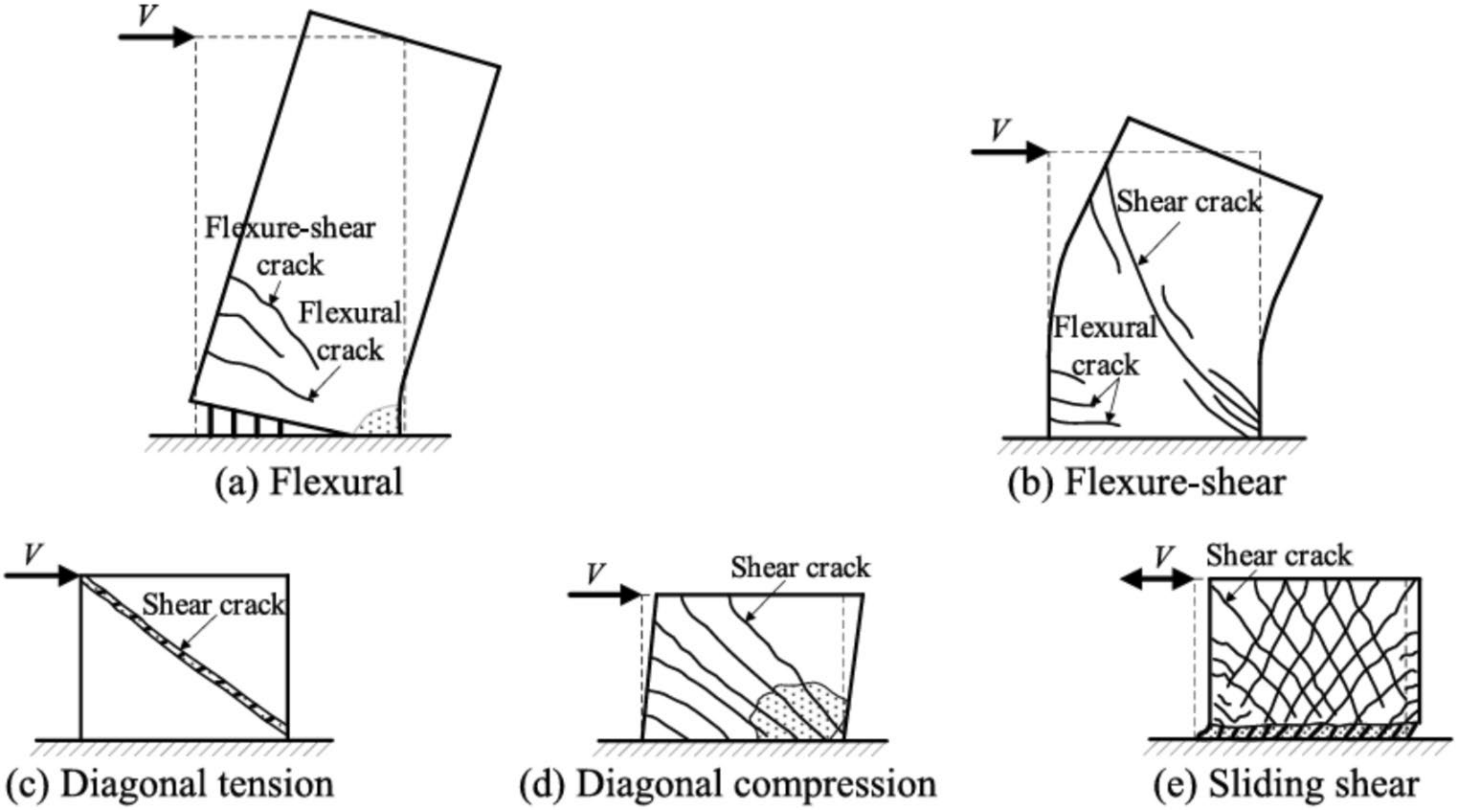
Failure modes of RC shear walls (Zhang et al.2022).

*Slender walls*, which are usually flexure-controlled, tend to experience flexural failure. This failure mode is characterized by cracking concrete in the plastic hinge region, yielding of the boundary longitudinal reinforcement, followed by either concrete crushing or fracture of the boundary bars, as illustrated in Fig. 2a. Flexural failure, however, is rarely observed in squat walls, particularly when the aspect ratio is below 1.0.

For moderate walls (1.5 ≤ h_x_/l_x_ ≤ 3.0), flexure–shear failure is commonly observed. This failure initiates flexural cracking in the plastic hinge region and yielding of the boundary longitudinal reinforcement, followed by yielding or fracture of the horizontal shear reinforcement, as shown in Fig. 2b. In this case, flexural yielding precedes the onset of shear failure at higher drift levels.

In contrast, squat walls with aspect ratios approximately less than 1.0–1.5 generally exhibit shear failure. Shear failure can be classified into three main types—diagonal tension failure, diagonal compression failure, and sliding shear failure depending largely on the amount of shear reinforcement.

- *Diagonal tension failure* occurs in walls with inadequate horizontal shear reinforcement and is characterized by corner-to-corner diagonal cracking and subsequent yielding or fracture of the horizontal reinforcement, as illustrated in Fig. 2c.
- *Diagonal compression failure* may develop when the diagonal compression struts crush in walls that have sufficient shear reinforcement but experience high average compressive stresses, as shown in Fig. 2d. Walls with boundary elements such as flanges or barbell ends are more susceptible to diagonal compression failure compared to rectangular walls. This is because these boundary configurations allow for greater reinforcement concentration at the wall ends, increasing flexural strength and consequently imposing higher shear demands on the web region.
- *Sliding Shear Failure* Often occurs due to yielding of vertical reinforcement, which opens cracks at the wall base, combined with concrete crushing spreading along the wall length. These mechanisms create a weak sliding surface under applied force or displacement reversal, as shown in Fig. 2e.

The sliding shear strength is primarily resisted by the kinking action of vertical reinforcement crossing the crack. To enhance resistance against this failure, diagonal reinforcements at the wall base are recommended.

### Recent advances in seismic performance assessment of RC structures

Recent researches have significantly broadened the understanding of seismic resilience and structural evaluation. Vyas, et al.^5^ demonstrated that incorporating the post-yield stiffness ratio into nonlinear analysis significantly improves the accuracy of seismic response predictions, leading to more uniform drift distributions. Similarly, Bush et al.^6^ investigated the efficiency of Yielding Brace Systems (YBS), revealing that their integration can increase base shear capacity from 0.30 to 0.75 and reduce drift demands by up to 53%. This was further supported by Vyas et al.^7^, who introduced a modified performance-based plastic design (MPBPD) that explicitly accounts for post-yield stiffness to ensure superior seismic reliability. Additionally, Bush et al.^8^ confirmed that YBS-equipped buildings of various heights maintain stable energy dissipation and higher reliability under severe demands. Beyond structural systems, Gondaliya et al.^9^ proposed an integrated GIS and machine learning framework for efficient large-scale vulnerability prioritization. The impact of geometric configurations has also been addressed; Bush et al.^10^ evaluated the influence of staggered shear wall openings, while Barser et al.^11^ optimized shear wall placements to mitigate torsional effects in asymmetrical buildings. Based on the above established concepts, an enhanced Fiber hinge modelling approach is employed in this paper to accurately capture the nonlinear flexural response of slender RC shear wall.

### Fiber hinge model

Fiber hinge model is a numerical approach for non-linear analysis, where cross-section of RC shear wall is discretized into smaller, uniaxial “fibers” (concrete and steel) to simulate distributed plasticity and material nonlinearities. When using a plastic hinge model inelasticity is being concentrated in a short hinge region. This is sufficient for the case where the axial loads in elements, such as beams, are negligible. While in fiber hinge model, inelasticity is distributed along the element length and nonlinear axial/flexural response of a wall is simulated using a series of uniaxial elements (or fibers).

In this model,the behaviour of the wall is defined directly through nonlinear material models for concrete and reinforcing steel, assuming that plane section before bending remains plane after bending. Each element (fiber) represents a small portion of the cross-section with assigned material stress–strain properties, enabling the model to capture cracking, yielding, and crushing accurately.

**Fig. 3:**
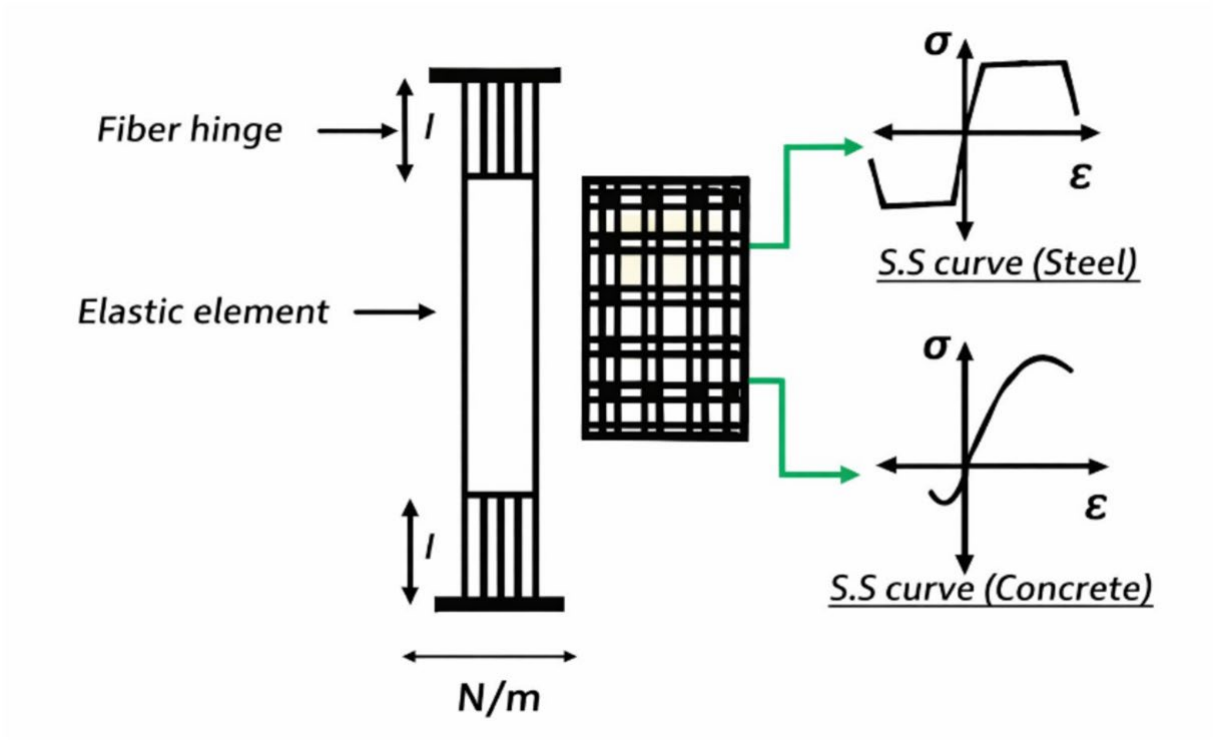
Fiber hinge modelling.

**Fig. 4:**
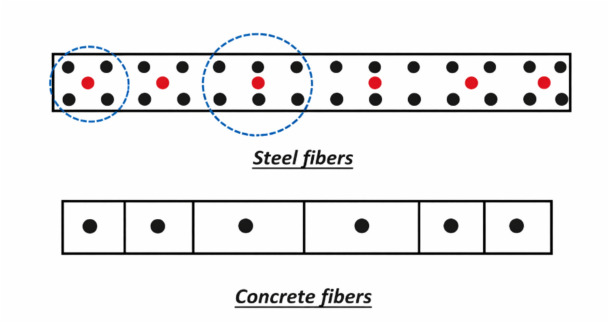
Fiber Hinge representation of wall: concrete and steel fibers.

Fiber hinge length can be defined to represent the portion of the wall where most of inelastic deformation or failure occurs. However, it is generally more accurate to model the full height of the wall using fiber elements to capture the overall deformation and stiffness degradation. Figures 3 and 4 illustrate the Fiber hinge approach:

### Material modelling

Accurate modelling of stress–strain behaviour of concrete and steel is a main factor in pushover analysis begins with understanding the compressive stress–strain behaviour of concrete in flexural members. Kent and Park^12^ conducted seminal research on flexural members with confined concrete, establishing that when the compression zone is confined by closely spaced steel stirrup ties, hoops, or spirals, the ductility of concrete is significantly enhanced, and large ultimate curvatures can be reached. Kent and Park^12^ used experimental results for compressive stress–strain curves of concrete confined by transverse steel to determine properties of the concrete compressive stress block at various strain levels. Kent-Park stress–strain model became base for subsequent research in concrete material modelling.

Kwak and Kim^13^ extended material nonlinear modelling to explicitly address loading behaviour through the development of stress–strain relations for concrete that account for material degradation. Although their work addressed cyclic loading mechanisms, the fundamental principles of capturing material nonlinearity through stress–strain relationships remain directly applicable to pushover analysis, where monotonic loading is applied incrementally until global failure occurs. The modifications to stress–strain relations reflecting average stress distribution in cracked RC elements—features essential for accurate nonlinear response prediction—apply to monotonic pushover loading sequences.

Ni et al.^14^ conducted an experimental study on five RC shear wall specimens under cyclic loading to investigate stiffness degradation behaviour. Their results showed that the constant reduction factors used in many design codes fail to capture the full degradation process of shear walls. To address this limitation, they proposed a four-line stiffness degradation model defined by four key stages: cracking, yielding, peak strength, and ultimate capacity. The study highlighted that higher reinforcement strength reduces initial stiffness, higher axial load ratios increase initial stiffness but accelerate degradation, flexural-type failures exhibit more complete stiffness loss than shear failures, and cross-sectional geometry strongly influences stiffness behaviour. The analytical stiffness curves predicted by their model closely matched experimental results, confirming the model’s validity for rectangular flexural walls and its applicability to pushover analysis.

Tang and Su^15^ critically examined the distinction between shear and flexural stiffness in RC shear walls, revealing that using gross shear stiffness—as adopted in many U.S. design codes—can overestimate actual shear stiffness by more than twofold. This overestimation affects predictions of building periods, load distribution, and seismic response accuracy. Their study emphasized that RC walls are governed by both shear and flexural deformation mechanisms, necessitating integrated modelling approaches. By comparing simplified analytical models and code recommendations (ACI 318–11, Eurocode 8, CSA) against experimental data, Tang and Su found that certain classical models can more accurately represent stiffness behaviour than overly conservative code values while remaining practical for engineering applications.

López et al.^16^ developed and validated the Efficient Shear-Flexure Interaction (E-SFI) model, a macro-modelling approach designed to accurately capture the nonlinear behaviour of planar RC walls while maintaining computational efficiency. The model introduces a calibrated expression for horizontal normal strain, simplifying the formulation to six degrees of freedom per element—similar to common Fiber-based models. Validation against 252 RC wall tests showed excellent agreement, with a predicted-to-experimental shear strength ratio of 1.04 and a coefficient of variation of 0.23. Further comparisons with ten detailed wall tests across varying shear span-to-depth ratios confirmed the model’s ability to accurately reproduce global, flexural, and shear responses, demonstrating its reliability in capturing shear-flexure interaction within RC walls.

Madani and Shahi^17^ developed a novel approach to estimate the stiffness and strength of damaged RC shear walls using surface crack patterns. This method addresses post-earthquake assessment needs by providing empirical relationships based on crack patterns extracted from images of damaged walls. By utilizing a database of images and employing symbolic and Bayesian regression^18^, a statistical method used to model relationships between variables, they propose predictive equations for residual strength and stiffness of damaged walls. This approach not only offers a quantitative method for assessing damage severity but also introduces a damage index based on crack patterns, which can facilitate validation of pushover analysis predictions against observed damage patterns in real structures.

Galal and El-Sokkary^19^ presented a comprehensive review of modelling techniques for RC shear walls, ranging from macro-models such as lumped plasticity and multi-axial spring models to micro-models including finite element and Fiber-based approaches. Their work identified critical challenges in modelling RC walls, including the combined effects of moment, shear, and axial forces, as well as bar slip, buckling, and boundary conditions.

Valoroso et al.^20^ developed a novel reinforced shell element for nonlinear pushover analysis that incorporates arbitrary distributions of steel reinforcement and nonlinear material behaviour for both concrete and rebars, demonstrating capabilities for obtaining limit loads and failure mechanisms at affordable computational cost.

The aforementioned of studies have represented their nonlinear behavior using analytical models, which have provided valuable insights into stiffness degradation and shear–flexure interaction. However, with the advancement of computational tools, numerical modeling has emerged as an efficient and reliable approach that can reproduce the complex response of RC elements with higher fidelity.

## Research significance

In this study, a numerical modelling framework is developed using finite element analysis with a fiber-hinge approach. This method enables direct incorporation of material nonlinearity, allowing a realistic simulation of stiffness degradation, strain localization, and shear–flexure interaction throughout the loading process. Compared to traditional analytical methods, the proposed numerical framework offers a more flexible and computationally efficient means to capture the progressive deterioration and post-peak behavior observed experimentally.

To enhance the conventional modelling strategy implemented in ETABS, this study addresses its key limitations and improves its capability to accurately represent the nonlinear behavior of shear walls under seismic loading. Experimental results from tested RC shear walls were collected and used to compare the performance of the conventional and proposed modelling strategies. The comparison was conducted through pushover analyses, and the resulting curves demonstrated that the proposed approach provides a practical and validated FEM methodology for RC shear walls governed by flexural, achieving an optimal balance between accuracy and computational efficiency. The modelling strategy incorporates advanced nonlinear constitutive models for both concrete and reinforcement and employs a refined mesh configuration to capture the global response while maintaining feasible computation times. The proposed approach integrates fracture-energy regularization with a four-line stiffness degradation model to improve numerical objectivity and reduce mesh sensitivity.

## Modeling of shear walls using conventional finite element modelling (CFEM)

The accuracy of the numerical model in capturing initial stiffness, yield point, peak load capacity, post-peak softening, and ultimate displacement capacity reflects its ability to simulate complex nonlinear phenomena such as stiffness degradation, strain localization, and the interaction between flexural and shear deformations. the model’s capability to replicate the experimentally observed progressive stiffness degradation, which is critical in understanding the seismic performance and failure mechanisms of RC shear walls, is crucial.

**Fig. 5:**
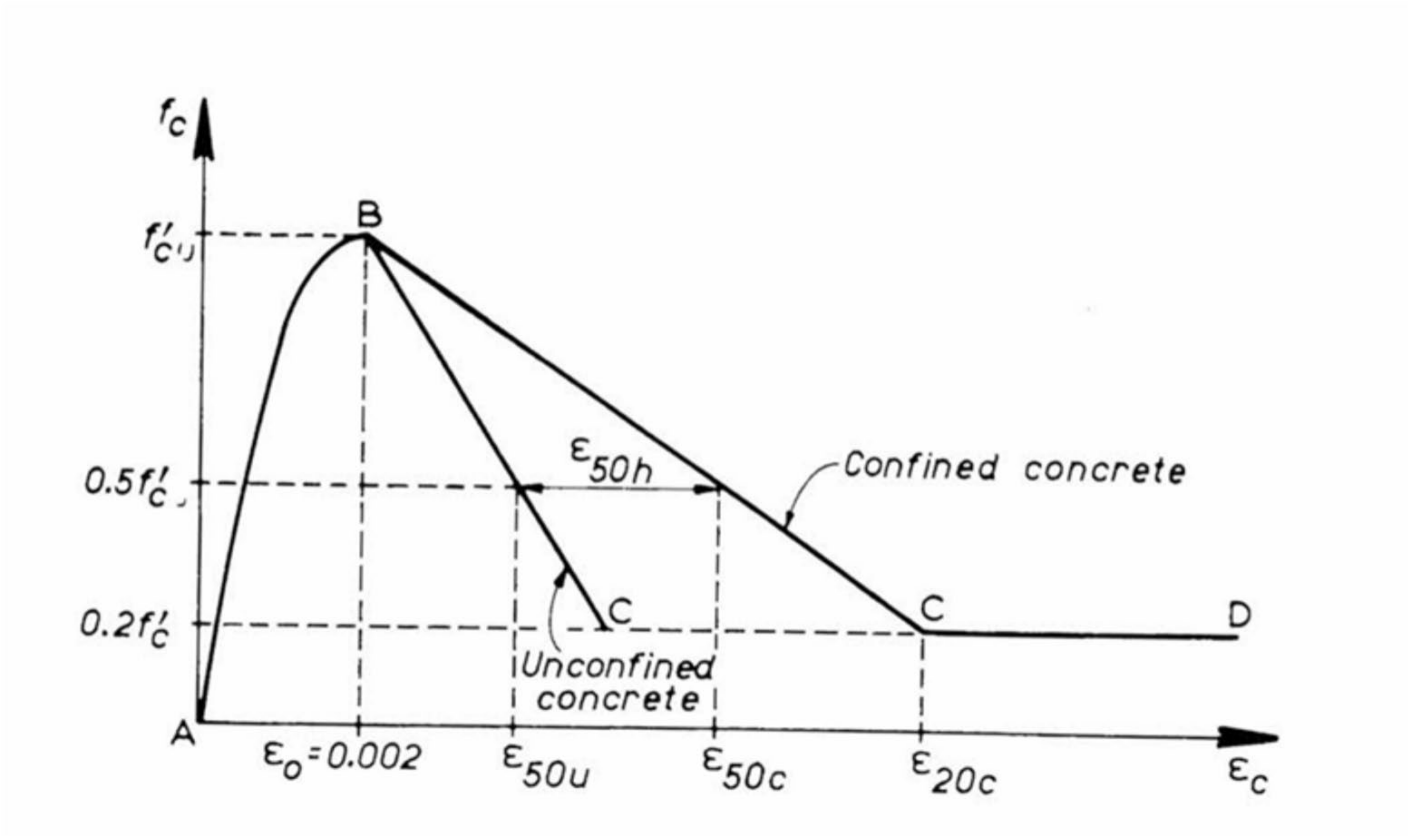
Kent-Park concrete stress–strain curve for confined and unconfined concrete^12^.

In this paper, RC shear walls specimens, obtained from previous experimental studies were modeled using ETABS software, employing the fiber hinge approach to capture the nonlinear behavior of the wall. Pushover analysis was conducted to evaluate the wall’s response under increasing lateral loads up to failure. Concrete was modeled using Kent-Park stress–strain relationship^12^ for confined concrete (Fig. 5), while the steel reinforcement’s stress–strain behavior was defined based on the material properties obtained from the experimental tests.

The whole wall was assigned to a Fiber hinge of type P-M3^21^ which accounts for combined axial force and biaxial bending effects.

The P-M3 formulation represents:

- **P**: Axial force behavior governed by axial stress–strain relationships of concrete and steel
- **M3**: Bending moment behavior in the plane perpendicular to the wall length, accounting for flexural degradation

**Fig. 6:**
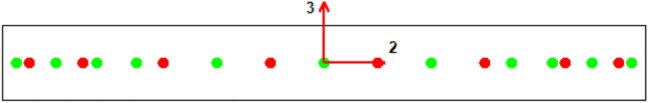
ETABS representation of the wall fibers in cross section (concrete fibers (in green) & steel fibers (in red)).

The shear wall was discretized into multiple small segments as shown in Fig. 6, the number and the boundaries of these segments is automatically generated in ETABS however, they can be modified if needed .At each load step, stress of each fiber was calculated based on current strain from stress–strain relationship. This discretization allows the model to capture nonlinear behavior within different regions of the wall under lateral loading.

### Verification of conventional finite element modelling (CFEM) with the experimental results

To verify the accuracy and reliability of the Conventional Finite Element Model (CFEM), its performance was validated against thirteen experimentally tested reinforced concrete walls. The key characteristics of these specimens are summarized from Table 1, 2, 3, 4, 5, 6, 7, 8 and Table 9 Each wall was subjected to quasi-static cyclic lateral loading using an actuator to simulate seismic effects and capture the inelastic behaviour of the structural system.

**Table 1:**
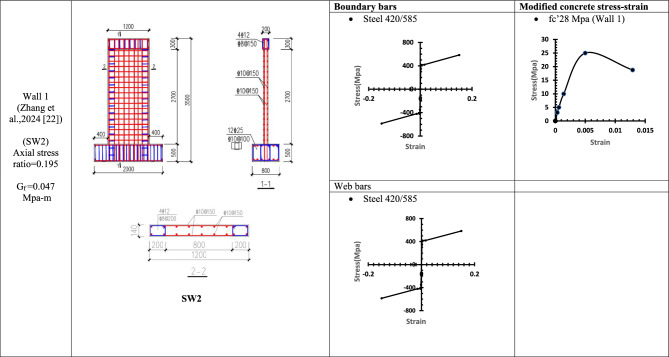
WALL 1 details^22^.

**Table 2:**
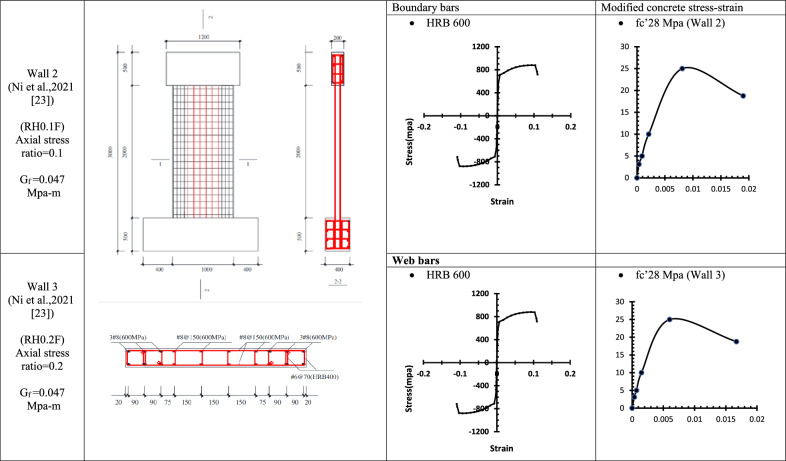
WALL 2 & WALL 3 DETAILS^23^.

**Table 3:**
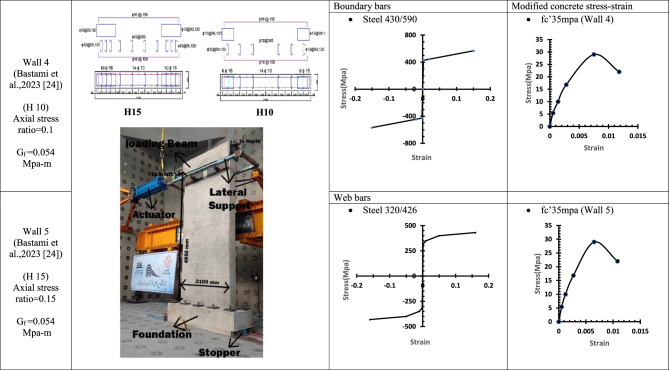
WALL 4 & WALL 5 DETAILS^24^.

**Table 4:**
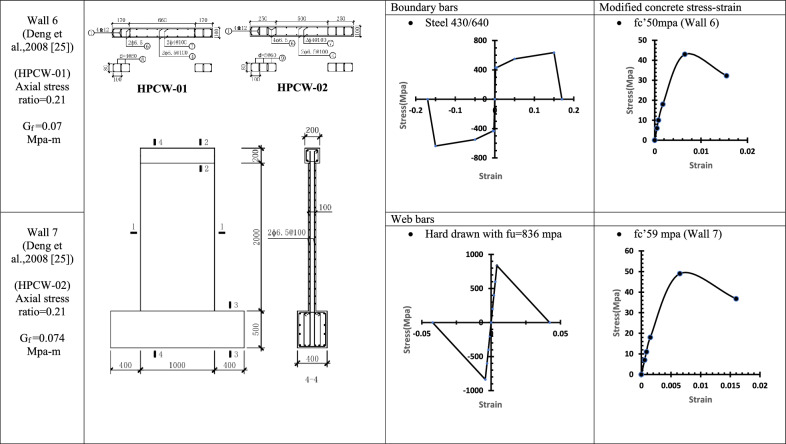
WALL 6 & WALL 7 details^25^.

**Table 5:**
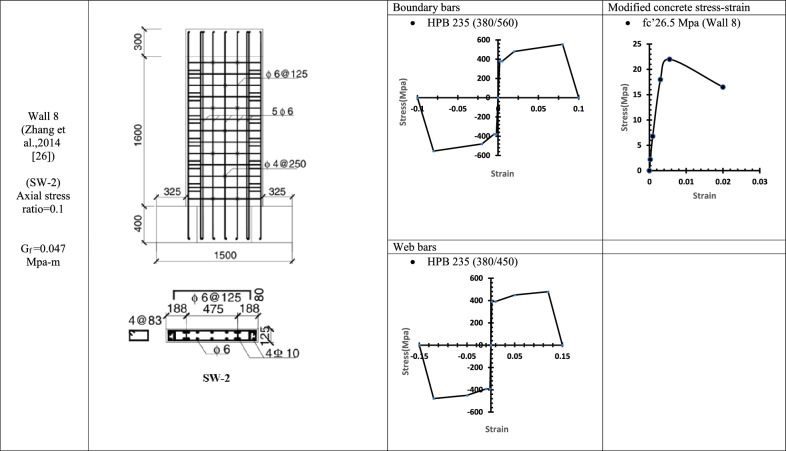
WALL 8 DETAILS^26^.

**Table 6:**
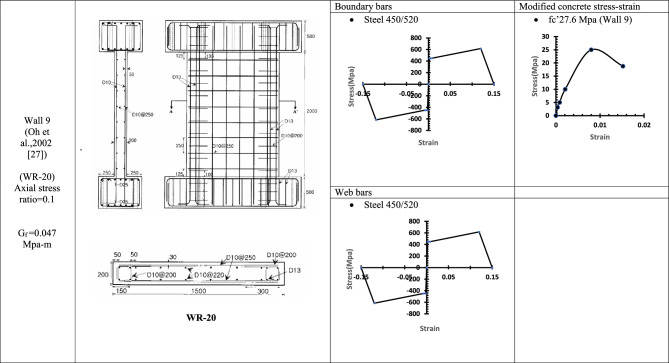
WALL 9 DETAILS^27^.

**Table 7:**
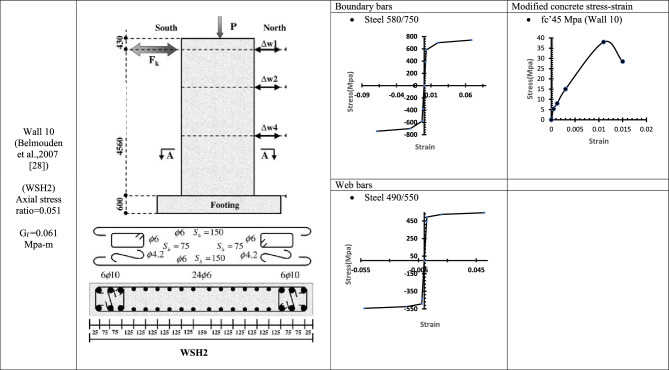
WALL 10 DETAILS^28^.

**Table 8:**
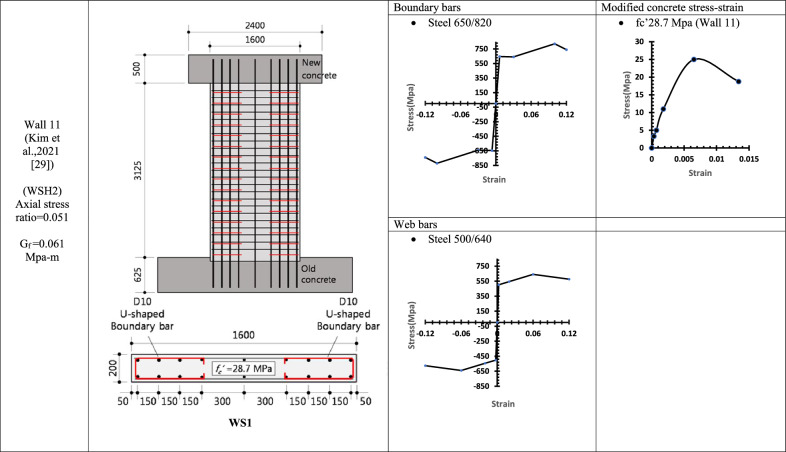
WALL 11 DETAILS^29^.

**Table 9:**
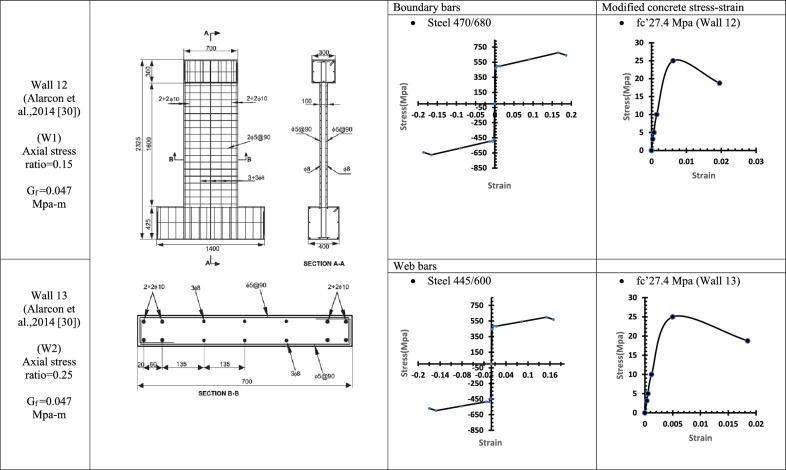
WALL 12 & WALL 13 DETAILS^30^.

**Fig. 7:**
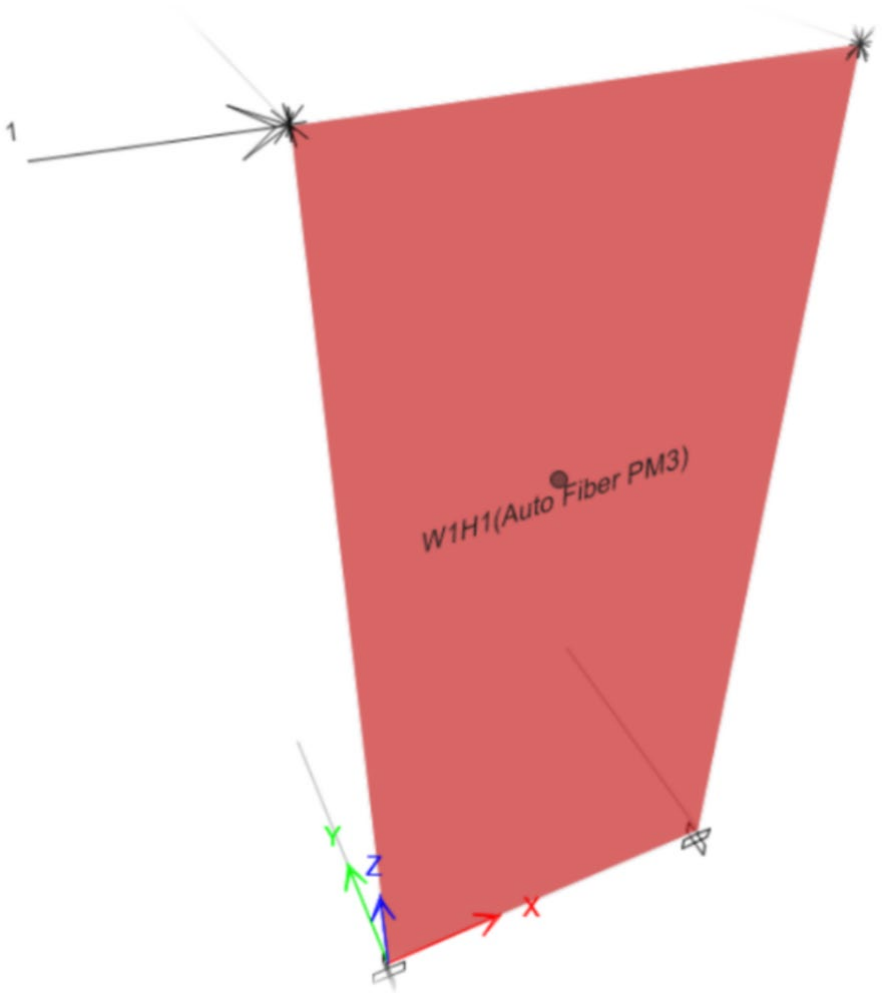
Load location and direction in deformation-controlled loading.

For numerical validation, a pushover analysis was performed on each corresponding finite element model to replicate the experimental setup. A unit lateral load was applied at the actuator’s location, as shown in Fig. 7, and the analysis was carried out under displacement-controlled loading conditions to enable precise tracking of post-yield behaviour.

The wall supports were modelled as fixed to represent the rigid connection between the wall footing and the laboratory’s strong floor. To simulate the actual test conditions where out-of-plane buckling was restrained by low-friction sliding bearings at the floor levels, out-of-plane movement is prevented in the model by applying roller supports in that direction and using 2D option in ETABS. This approach ensures that no displacement occurs out of plane, effectively replicating the experimental boundary conditions and ensuring consistency with the experimental boundary conditions.

**Fig. 8:**
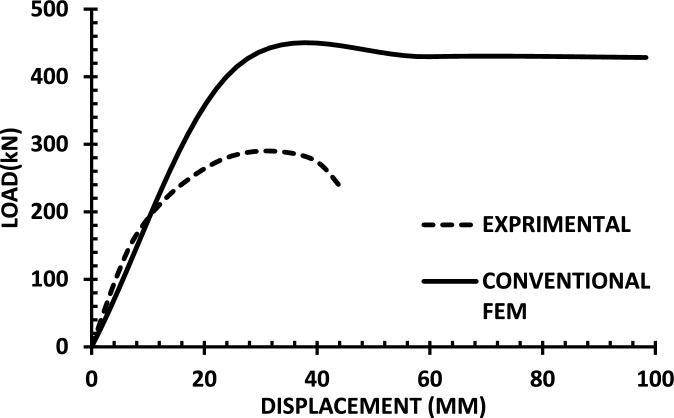
Wall 1 Experimental vs Conventional FEM numerical results (SW2- Zhang et al.^22^).

**Fig. 9:**
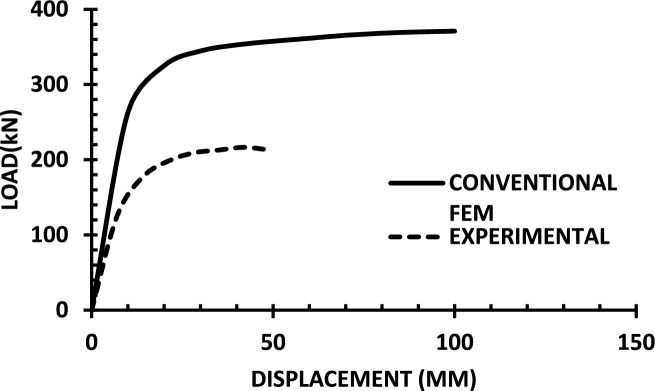
Wall 2 Experimental vs Conventional FEM numerical results (RH0.1F- Ni et al.^23^).

**Fig. 10:**
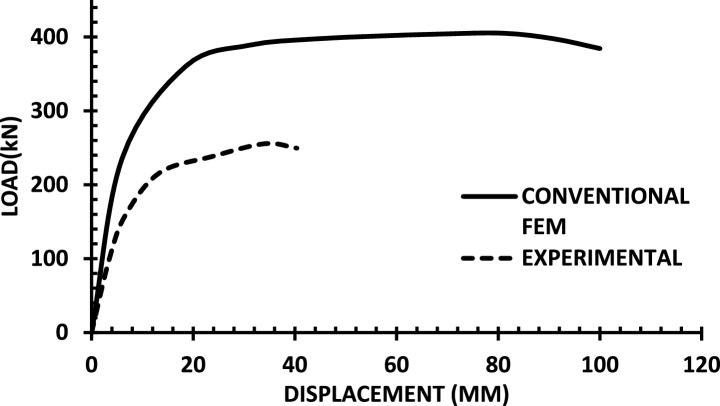
Wall 3 Experimental vs Conventional FEM numerical results (RH0.2F- Ni et al.^23^.

**Fig. 11:**
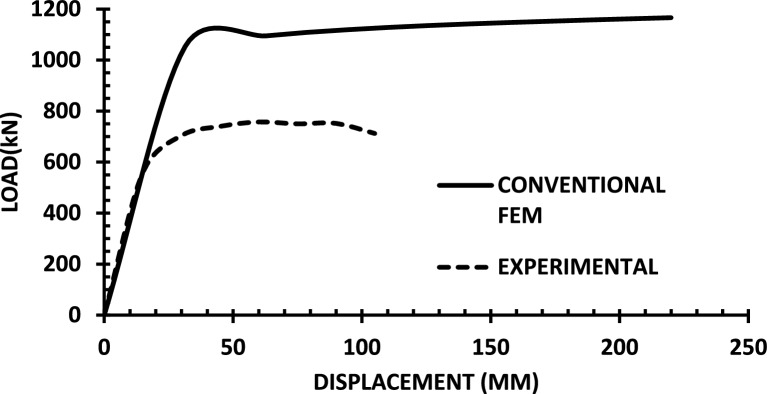
Wall 4 Experimental vs Conventional FEM numerical results (H 10-Bastami et al.^24^).

**Fig. 12:**
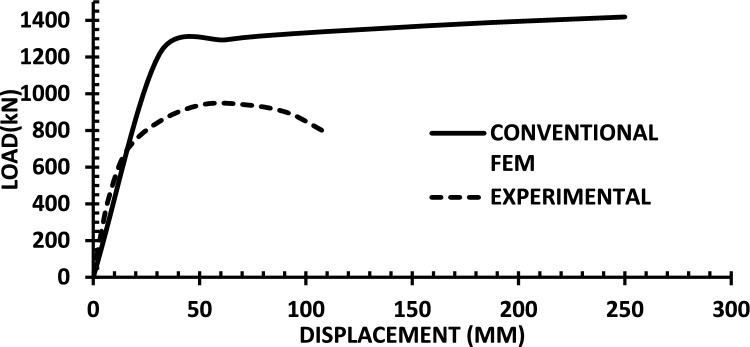
Wall 5 Experimental vs Conventional FEM numerical results (H 15-Bastami et al.^24^).

**Fig. 13:**
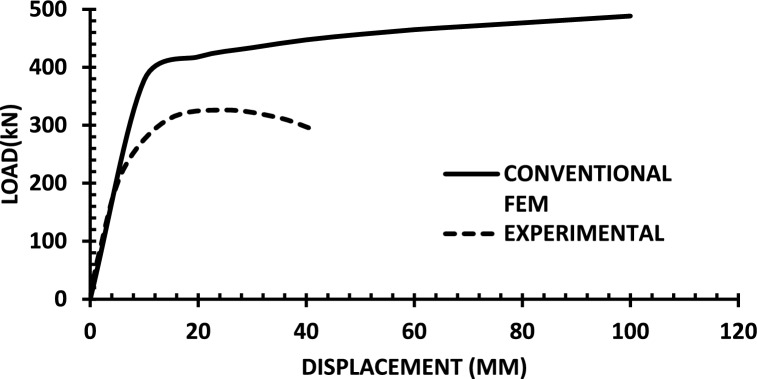
Wall 6 Experimental vs Conventional FEM numerical results (HPCW-01-Deng et al.^25^).

**Fig. 14:**
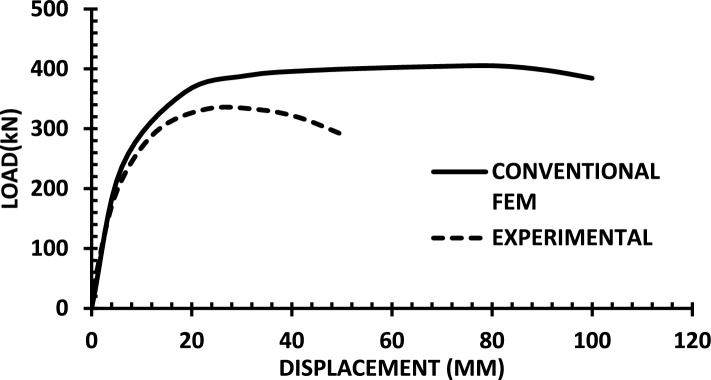
Wall 7 Experimental vs Conventional FEM numerical results (HPCW-02-Deng et al.^25^).

**Fig. 15:**
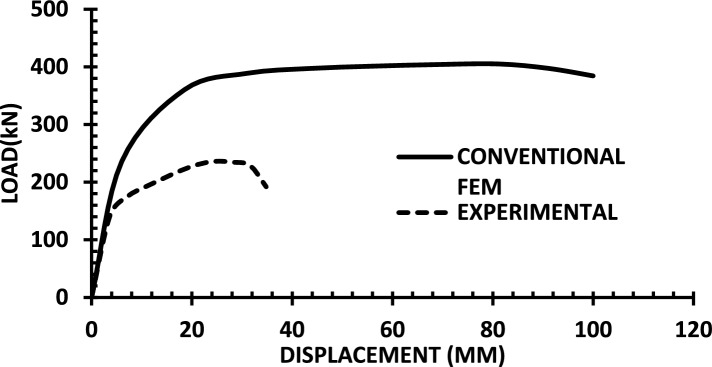
Wall 8 Experimental vs Conventional FEM numerical results (SW-2-Zhang et al.^26^).

**Fig. 16:**
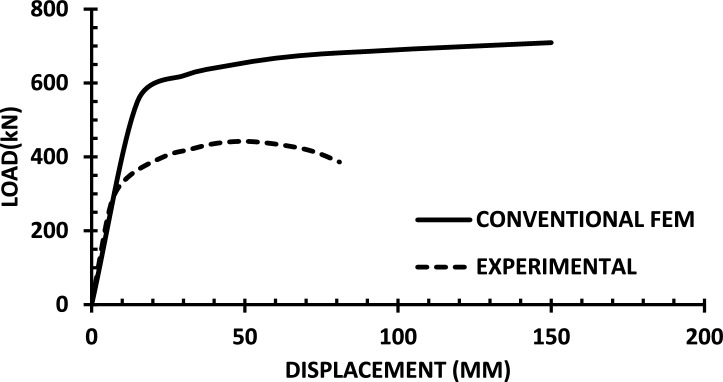
Wall 9 Experimental vs Conventional FEM numerical results (WR-20-Oh et al.^27^).

**Fig. 17:**
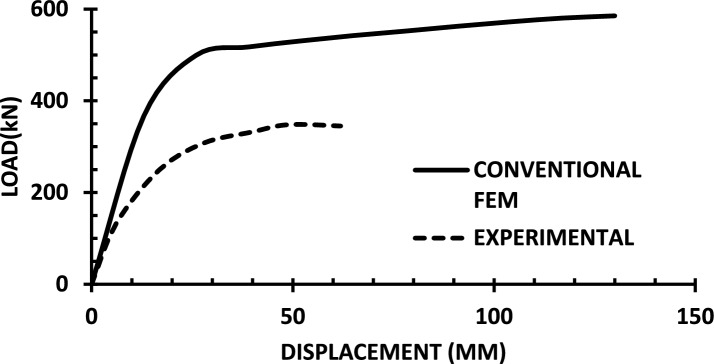
Wall 10 Experimental vs Conventional FEM numerical results (WSH2-Belmouden et al.^28^).

**Fig. 18:**
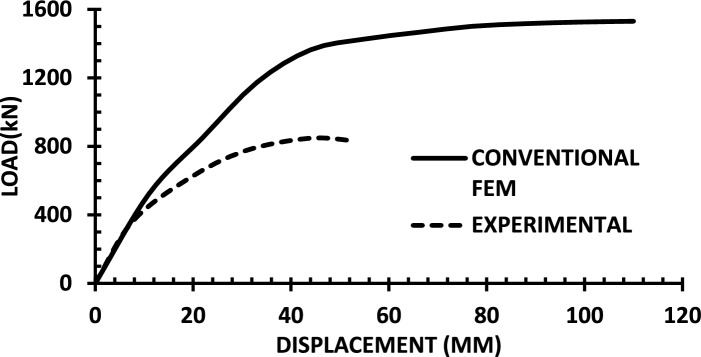
Wall 11 Experimental vs Conventional FEM numerical results (WSH2-Kim et al.^29^).

**Fig. 19:**
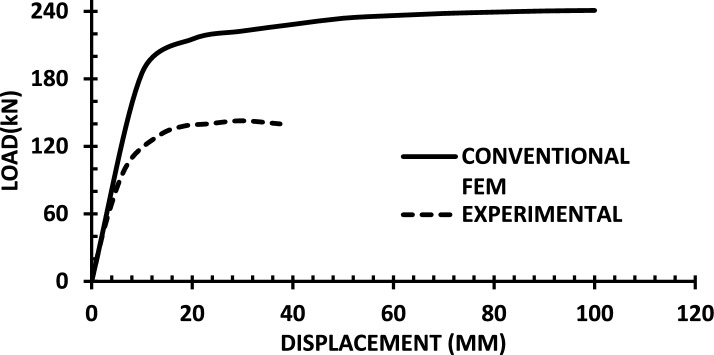
Wall 12 Experimental vs Conventional FEM numerical results (W1-Alarcon et al.^30^).

**Fig. 20:**
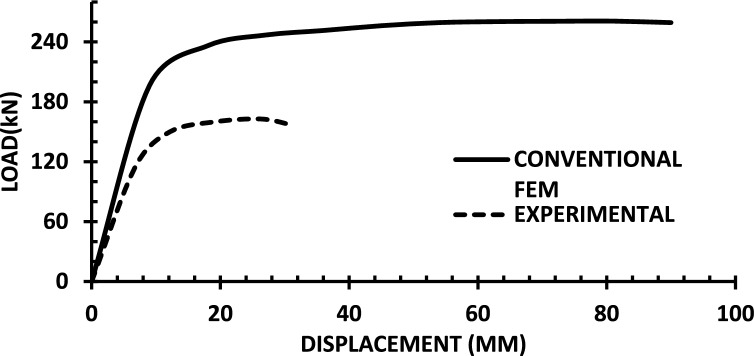
Wall 13 Experimental vs Conventional FEM numerical results (W2-Alarcon et al.^30^).

The resulting pushover curves from the numerical models were compared with the experimental load–displacement responses, as shown in Figs. 8, 9, 10, 11, 12, 13, 14, 15, 16, 17, 18, 19, 20. The comparison showed notable discrepancies between the numerical and experimental results, especially in the post-yield phase, with differences observed in both strength and displacement capacities. These findings indicate that the conventional finite element model does not fully replicate the experimental behaviour, emphasizing the need for further refinement.

## Problems associated with CFEM

The previous discussed model has limitations in accurately assessing the capacity, as demonstrated by the verification study, where results showed excessive energy dissipation and significant discrepancies between experimental tests and finite element method (FEM) outcomes. This section addresses the challenges and limitations of the model, highlighting areas where improvements are needed to enhance its predictive accuracy.

Main challenges in modelling concrete shear walls using fiber hinges:

- The localization of concrete failure.
- Reduction in wall stiffness resulting from the influence of shear deformations.

### Localization of concrete failure

**Fig. 21:**
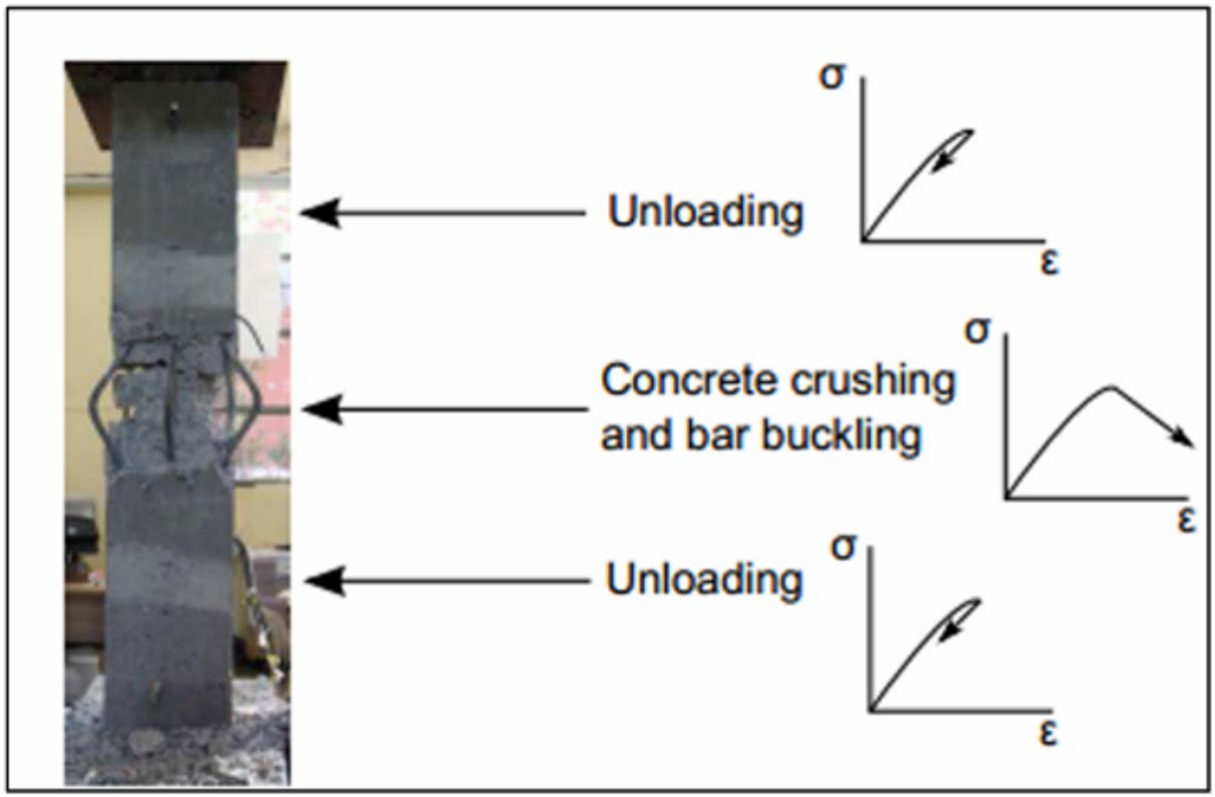
Localization under pure axial load (Massone et al. 2014).

Localization of concrete failure represents a fundamental challenge in Fiber-based element modeling. When an element is subjected to uniform compression, all fibers and sections experience identical strain and stress states under equal loading. However, concrete exhibits strain softening after reaching maximum strength through crushing. Under such conditions, deformations tend to concentrate at the first section reaching softening behavior, the weakest link, while other sections unload to maintain axial equilibrium. This phenomenon is experimentally observed in all softening materials, such as concrete, as shown in Fig. 21.

In flexibility-based fiber element models^31^, the numerical manifestation of localization behavior appears as a dependency of structural response on the number of integration points used along the element length. Integration points distributed over the element length define the segment length associated with each cross-sectional analysis point according to the numerical integration scheme. The fracture zone where concrete crushing occurs, corresponds to one of these segment lengths. Mathematically, this localization dependency can be expressed as:

1 $$\Delta u=\sum \left({L}_{i}\times {\upvarepsilon}_{i}\right)=\sum \left({L}_{i}\times 0\right)+{L}_{d}\times {\varepsilon}_{d}$$

**Fig. 22:**
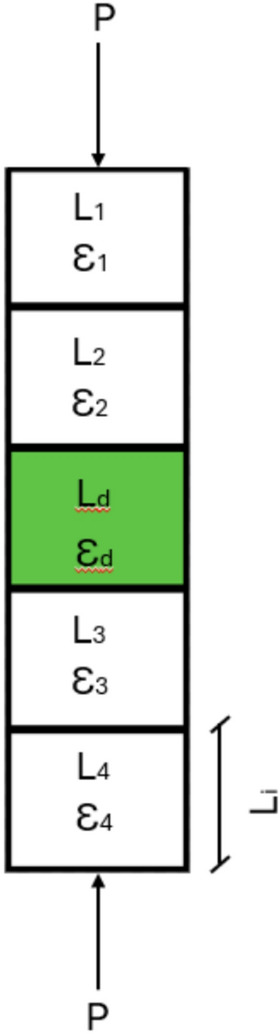
Finite element model represents the integration points length with their corresponding strains.

As illustrated in Fig. 22 L_i_ and ${\varepsilon}_{i}$ represent the length and corresponding strain associated with each integration point, respectively, while ${L}_{d}$ and ${\varepsilon}_{d}$ denote the length and strain of the damaged zone. This expression clearly illustrates that the computed global deformation is influenced by the discretization length and strain distribution assigned to the integration points, leading to non-objective model results that depend on mesh discretization rather than the actual material behavior.

**Fig. 23:**
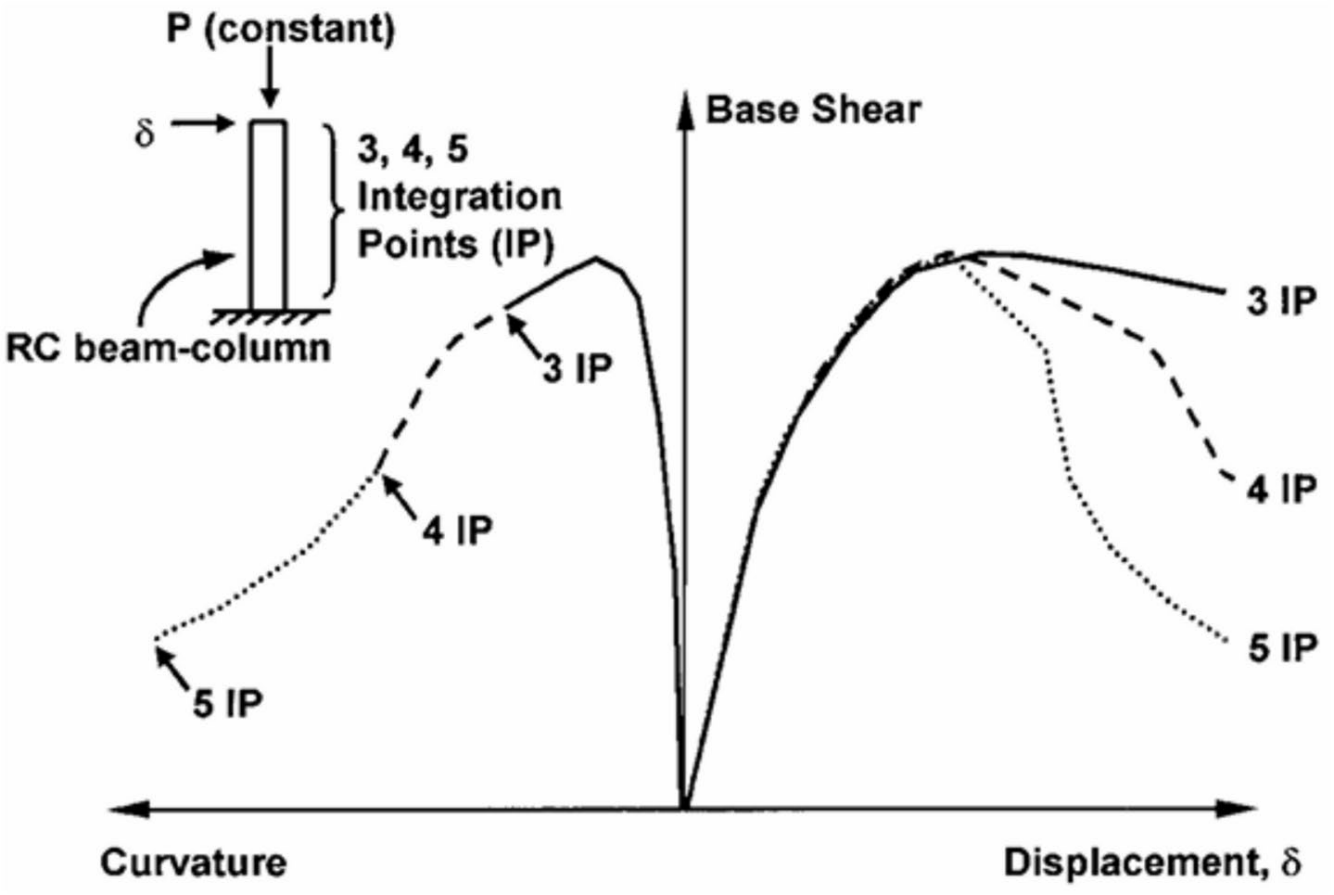
RC Beam-Column Modeled with Strain-Softening Section Response^33^.

**Fig. 24:**
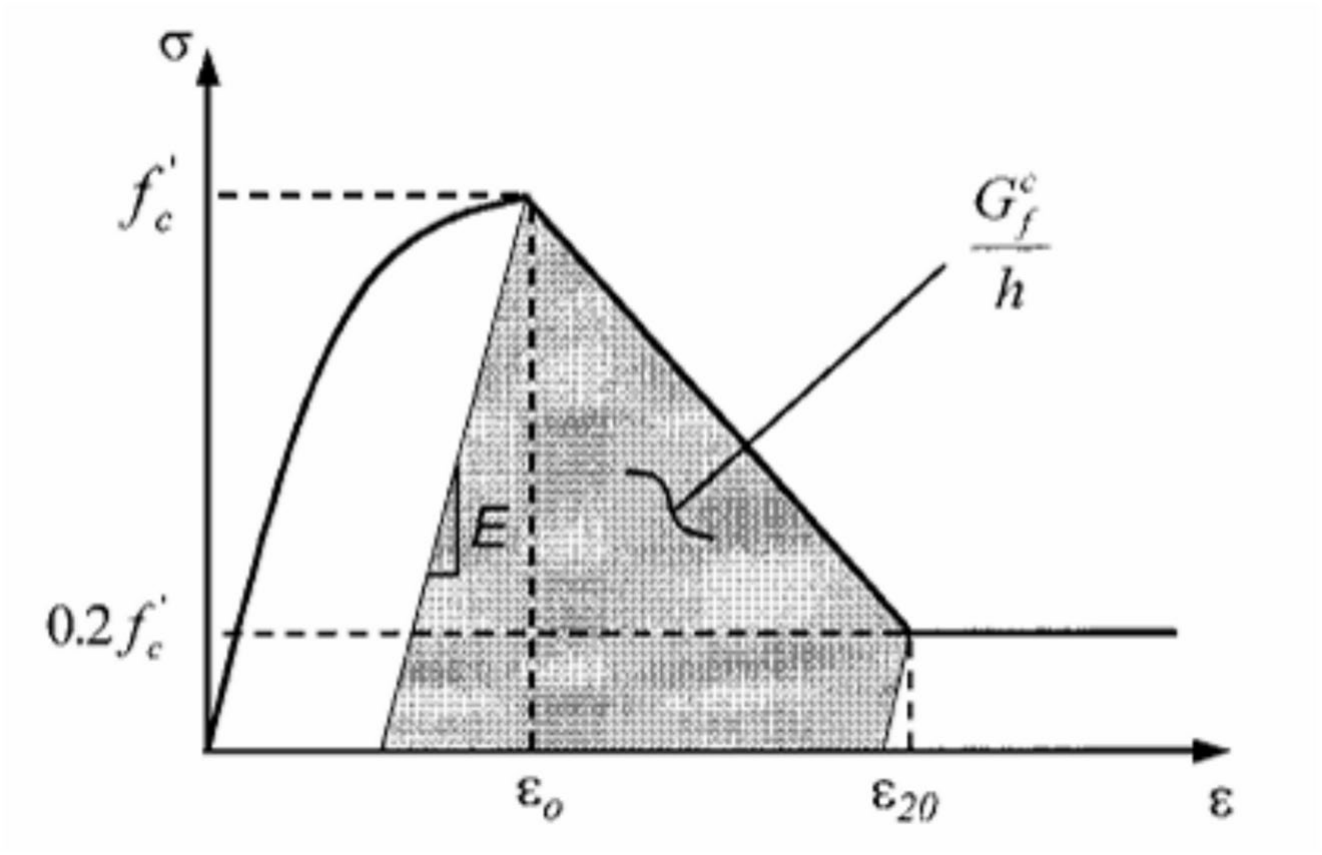
Modified Kent-Park (1971) Stress–Strain Law and Compression Fracture^33^.

Jansen and Shah^32^ demonstrated through experimental studies that compressive strain softening in concrete is a localized phenomenon, where post-peak stress–strain behaviour depends on the specimen length, whereas the fracture energy remains an intrinsic material property independent of geometry. Their findings highlighted the need for modelling approaches that explicitly consider localization to ensure objective material representation. Building on this foundation, Coleman and Spacone^33^ conducted experimental and numerical investigations on RC beam-columns subjected to lateral displacement under axial load, as shown in Fig. 23. They observed that load–displacement and curvature responses were highly sensitive to the number of integration points, revealing the non-objective nature of conventional softening constitutive models. To overcome this limitation, they introduced a fracture energy–based regularization technique, modifying the descending branch of the Kent–Park model shown in Fig. 5 so that the area under the curve equals the concrete’s fracture energy divided by the integration length. This ensured mesh-independent finite element results governed by material properties rather than discretization. The regularization procedure involves modifying the post-peak stress–strain relationship by adjusting the slope and extent of the descending branch, as shown in Fig. 24

2 $$Area under the Desceding branch={G}_{f} /h$$

where fc′ is the concrete compressive strength, and h is the characteristic damage zone length equal to the integration segment length. Experimental calibration by Lopez et al. indicates that for concrete compressive strengths ranging from 23 to 40 MPa, the fracture energy ranges from 0.039 MPa.m to 0.056 MPa.m. By incorporating this fracture energy-based regularization into the Kent-Park stress–strain curve, finite element predictions become mesh-independent, with computed responses determined by material properties rather than discretization choices.

### Reduction in wall stiffness resulting from the influence of shear deformations

**Fig. 25:**
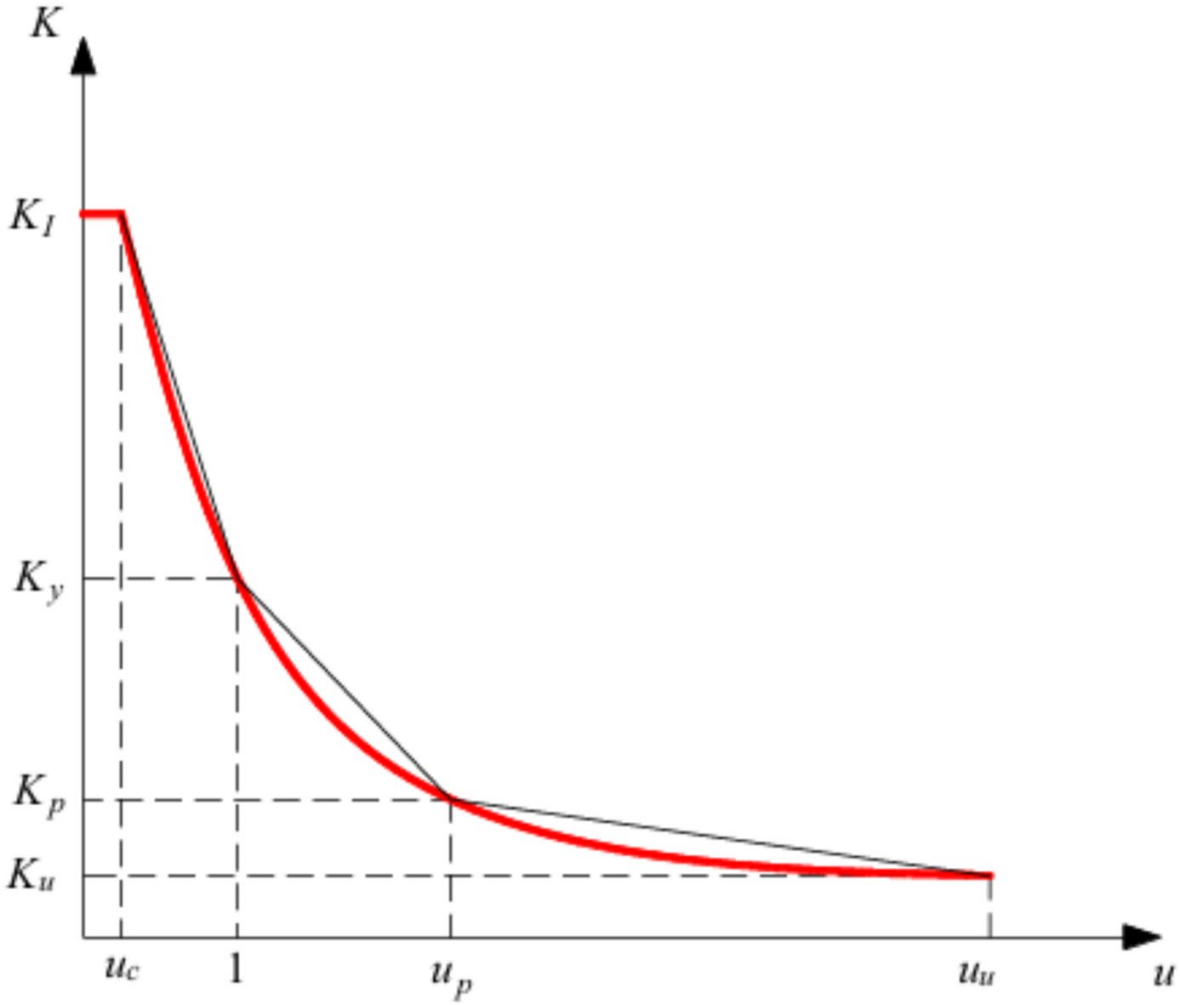
Four-line stiffness degradation model of rectangular shear walls failing in flexure^14^.

**Fig. 26:**
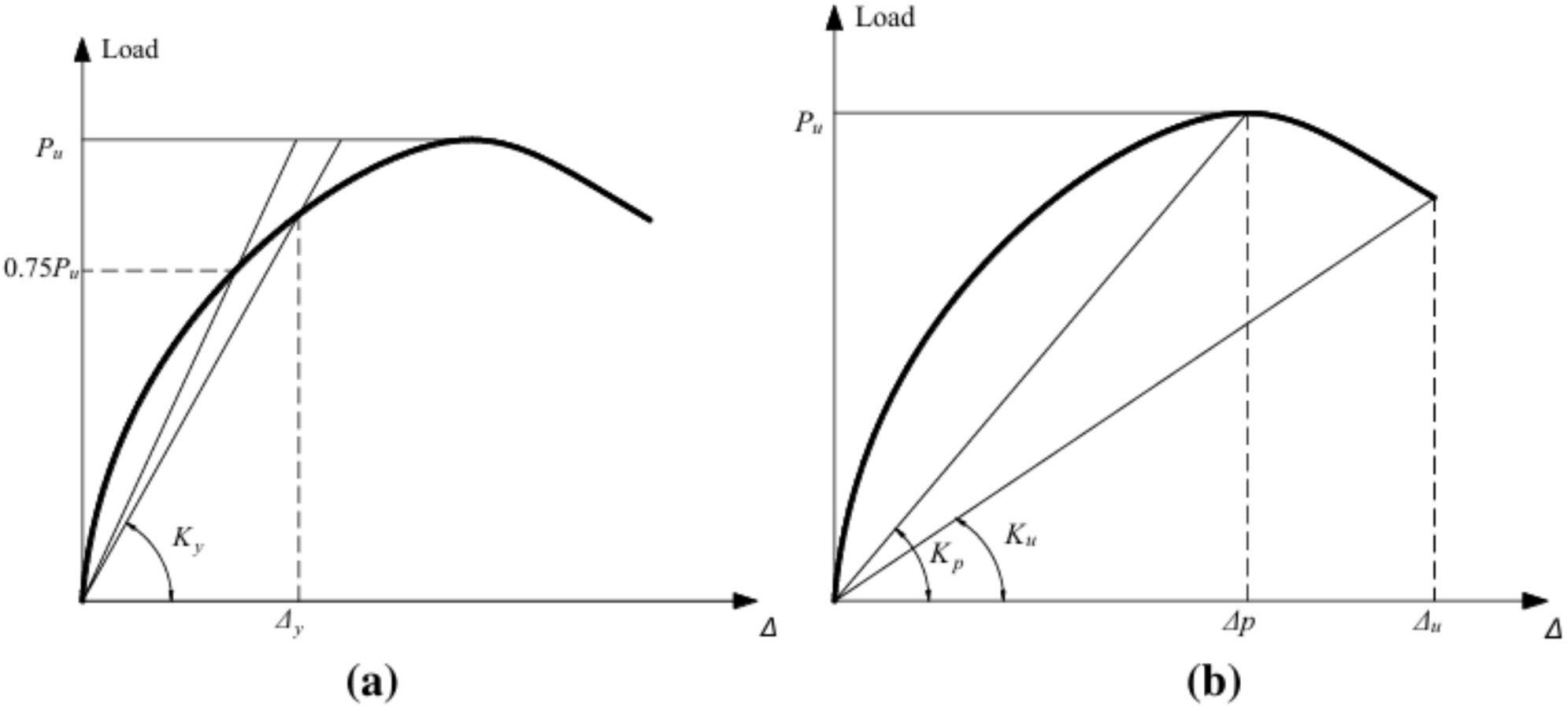
Definition of feature points: a yield stiffness, b peak and ultimate stiffness^14^.

To address progressive stiffness degradation under lateral loading, Ni et al.^14^ proposed a four-line stiffness degradation model for reinforced concrete shear walls failing in flexure, as shown in Fig. 25. The model is governed by four critical points, crack initiation, yield, peak strength, and ultimate capacity, each defining a distinct phase of the loading envelope. This formulation captures the nonlinear progression of stiffness degradation due to combined shear and flexure effect observed experimentally by assigning specific stiffness characteristics to each stage. As loading progresses, the model transitions smoothly from initial to ultimate states, accurately representing the experimentally observed degradation behavior of rectangular shear walls governed by flexural response. Validation of the model confirmed its applicability to walls subjected to lateral loading under constant axial load, with height-to-width ratios ≥ 1.7, axial loads below 0.62f′c, concrete strengths between 21.43 MPa and 73.21 MPa, and reinforcing bar strengths between 362 and 1044 MPa.

where K_I_ is the initial stiffness of the shear walls, uc is the crack displacement ductility, K_y_ is the yield stiffness, K_p_ is the peak stiffness, u_p_ is the peak displacement ductility, Ku is the ultimate stiffness and u_u_ is the ultimate displacement ductility (Fig. 26).

Initial Stiffness (K_i_) at uncracked condition is computed as:

3 $${K}_{i}={( ( {H}^{3}/{c}_{1}3{E}_{c}{I}_{c})+(H/ {c}_{2}{G}_{c}{A}_{w}) )}^{-1}$$

4 $${I}_{c}=\left(1+({E}_{s}/{E}_{c}) {\rho}_{s}\right) b{{h}_{w}}^{3} /12$$

5 $${\rho}_{s}=As+({E}_{sw}/{E}_{s}){A}_{sw}/b{h}_{w}$$

where E_c_​ is the elastic modulus of concrete, E_sw_ is the elastic modulus of the vertical distribution reinforcement in the non-confined area of the cross-section, Gc is the shear modulus of concrete, H is the shear wall height, A_w_ is the cross-sectional area, I_c_ is the equivalent moment of inertia accounting for reinforcement effects, and the second term represents shear deformation contribution. Cracking factors c1 and c2 (0.35 and 1.0, respectively, per ACI 318-14) modify concrete properties to account for anticipated cracking.

Yield Stiffness (Ky) is calculated as:

6 $${K}_{y}=3{E}_{c}{I}_{y}/{H}^{3}$$

7 $${I}_{y}=0.172\left(1+4.4(N/fc{\prime}A)\right)\left(0.62+(190/ {f}_{y})\right)\left(0.76+0.005fc{\prime}\right){I}_{c}$$

where I_y_ is the conversion section moment of inertia of the yield stiffness, K_y_ is the effective stiffness, fc is the concrete compressive strength of cylinders, and f_y_ is the yield strength of vertical reinforcing bar.

Peak Stiffness (K_p_) is determined from experimental moment–curvature analysis at peak strength:

8 $${K}_{p}=0.54{K}_{y}$$

Ultimate Stiffness (K_u_) is computed at ultimate deformation capacity:

9 $${K}_{u}=0.3{K}_{y}$$

## Proposed FEM methodology

**Fig. 27:**
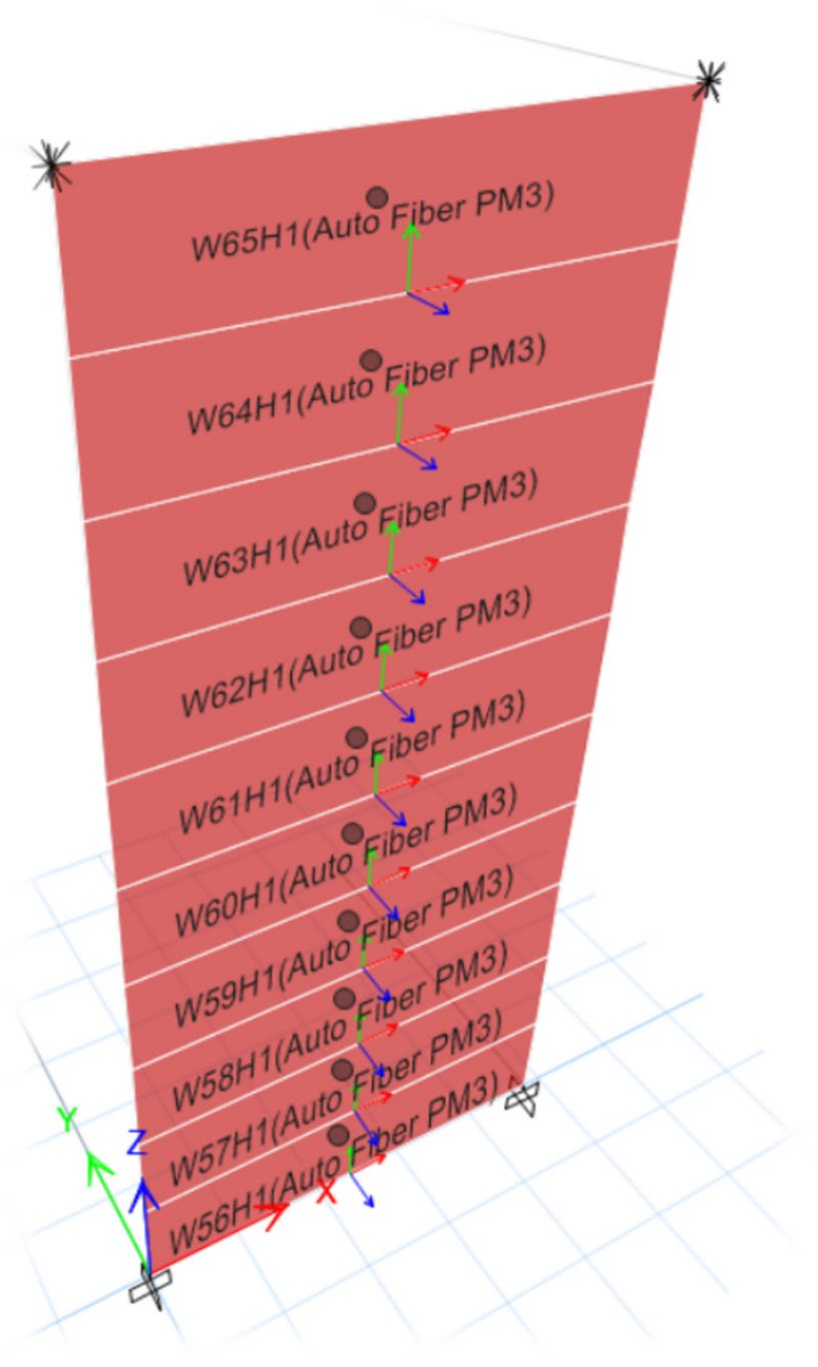
Proposed Fiber hinge assignment for the studied walls.

The first step in the Fiber-hinge analysis involves discretizing each RC shear wall into a definite number of segments while keeping the area under the descending branch for Kent and Park stress–strain curve the same. In this study, ten segments along its height were proposed, as shown in Fig. 27, while modified stress–strain curve of concrete is shown in Fig. 29 This discretization defines the integration points for the Fiber analysis, allowing proper simulation of cracking and material degradation along the wall height.

The discretization length L_d_​ associated with each segment directly controls the fracture energy regularization applied to the concrete stress–strain curve. With ten segments, the integration point spacing ensures adequate representation of concentrated deformation zones while maintaining computational tractability.

**Fig. 28:**
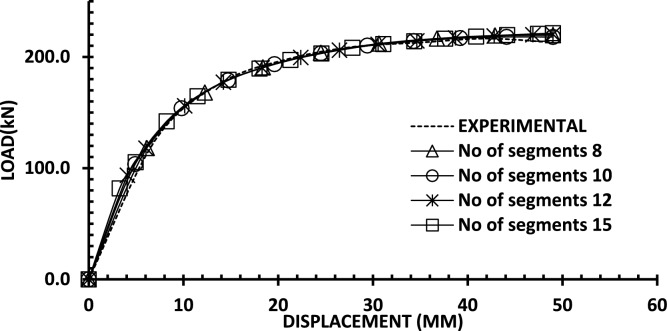
Comparison of mesh independence using fracture energy regularization for Wall 2.

To investigate mesh sensitivity in alignment with fracture energy regularization, a convergence study was conducted for wall 2 using 8, 10, 12, and 15 segments. The global response remains stable across all cases, as illustrated in Fig. 28. This consistency is due to the fracture energy regularization, which adjusts the concrete softening behavior based on the segment size. Thus, 10 segments were selected as an optimal choice for both accuracy and efficiency .

The second step is modifying the concrete stress–strain curve by implementing the four-line stiffness degradation model within the finite element framework. This involves progressively adjusting Young’s modulus (E) at key points along the curve, crack initiation, yield, peak, and ultimate, to accurately represent the element stiffness at each loading stage. This modification ensures the stress–strain behaviour implicitly captures the progressive stiffness degradation observed experimentally.

**Fig. 29:**
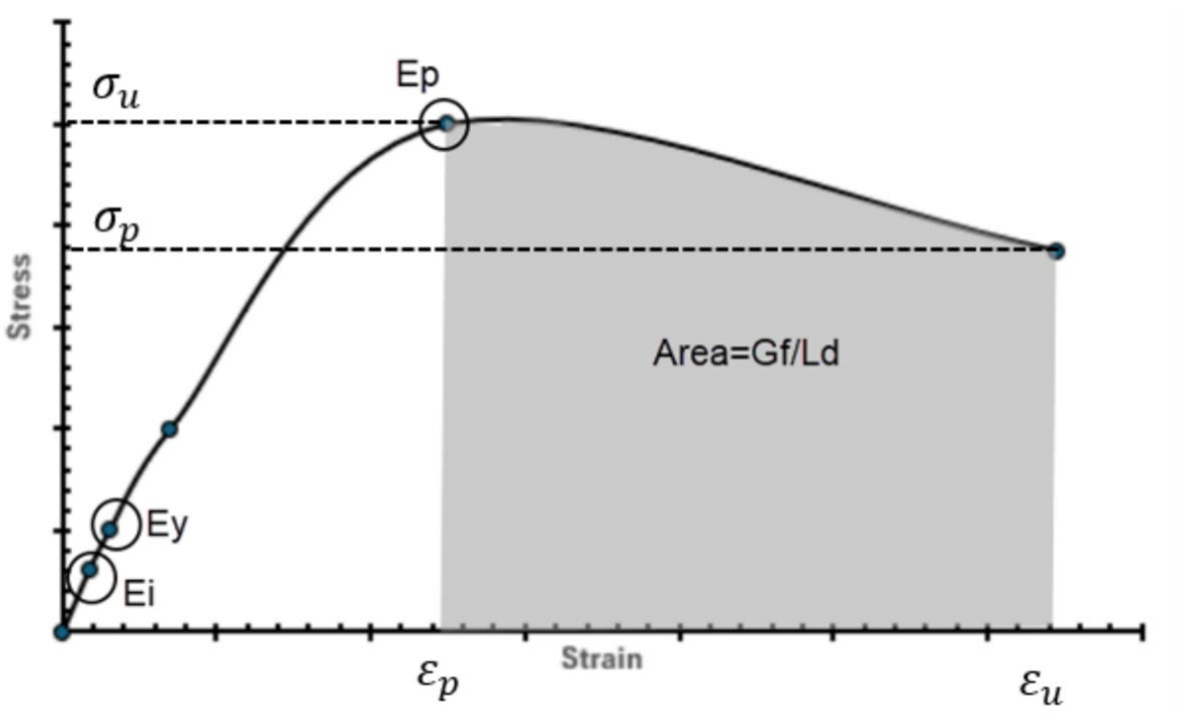
Modified stress–strain curve of concrete.

Figure 29 shows the modified stress–strain curve of concrete, While Equations from 10 to 14 illustrate the mathematical formulation used to determine its control points.

10 $${E}_{i}=\left({K}_{i}/ {K}_{o}\right){E}_{c}$$

11 $${E}_{y}=\left( {K}_{y}/{K}_{o}\right){E}_{c}$$

12 $${E}_{p} =\left({K}_{p}/{K}_{o}\right){E}_{c}$$

13 $${E}_{c}=4700\sqrt{fc{\prime}}$$

14 $${K}_{o} =3{E}_{c} {I}_{c}/{H}^{3}$$

In each segment transition, the slope of the stress–strain curve is adjusted such that the corresponding secant stiffness matches the four-line model stiffness values. This mathematical linkage ensures that finite element predictions using the modified stress–strain curve reproduce the four-line stiffness degradation model implicitly through material constitutive behavior rather than explicitly through stiffness reduction algorithms.

The modified stress–strain curves incorporate fracture energy-based regularization in the descending (softening) branch to ensure mesh-independent results. The descending branch from peak stress to residual stress is modified such that:

15 $${G}_{f}=Ld*\left(({\sigma}_{u} +{\sigma}_{p})/2 \right)\left({\varepsilon}_{u}-{\varepsilon}_{p}\right)$$

where L_d​_ is the integration segment length. This ensures that the model response reflects material properties and four-line stiffness degradation rather than numerical discretization.

In the proposed model, solved iterations showed that the ultimate stress (σ_u_) is about equal to 0.8 f.′c, while the failure stress (σ_p_) is equal to 75% of the ultimate stress (σ_p_ = 0.75 σ_u_). The corresponding strain is then determined using Eq. (15) to achieve the target fracture energy.

The steel stress–strain relationship in this study is directly defined based on experimental data for both the boundary and web reinforcement, ensuring accurate representation of material behavior. The boundary reinforcement curve captures the confined behavior and enhanced ductility of the longitudinal bars located in the wall boundaries, whereas the web reinforcement follows the stress–strain relationship corresponding to the distributed vertical bars in the unconfined region.

By integrating the modified stress–strain relationship of concrete, including the adjusted Young’s modulus and fracture energy parameters, the proposed model provides a more realistic representation of material softening and failure progression. Consequently, the finite element model demonstrates improved agreement with experimental results and more accurately reproduces the overall pushover response of the tested walls.

## Verifications of the proposed FEM methodology with experimental data

To ensure the accuracy and reliability of the proposed finite element model to accurately reproduce key experimental responses such as load displacement behavior, stiffness degradation, and ultimate strength under testing condition, the developed model is verified against. The aforementioned thirteen reinforced concrete shear wall specimens-previously detailed in the CFEM section- were used as the benchmark for validation. These experimental programs were selected to represent a wide range of wall configurations, including variations in geometry, aspect ratio, axial load level, reinforcement detailing, and failure mode. It is important to note that while the experimental walls were tested under cyclic loading, the proposed finite element model was analysed using a monotonic pushover approach.

The parameters and modified concrete stress-strain curves for these walls remains as presented from Table 1, 2, 3, 4, 5, 6, 7, 8 and Table 9. 

## Results and discussion

In this section, the numerical results obtained from the proposed finite element model are compared with the experimental data for the thirteen reinforced concrete (RC) shear walls selected from nine independent studies. The comparison primarily focuses on the load–displacement behaviour, which serves as a fundamental indicator of the global structural response under lateral loading. The load–displacement curves provide valuable insight into multiple key response characteristics, including the initial stiffness, yield point, peak load capacity, post-peak softening, and ultimate displacement capacity.

It should be noted that the experimental results were obtained from cyclic loading tests, whereas the numerical simulations in the present study were performed using monotonic pushover analysis. Consequently, certain cyclic response characteristics such as stiffness degradation during unloading–reloading cycles, pinching behaviour, cumulative damage, and energy dissipation cannot be fully captured by the monotonic analysis adopted in this study. Nevertheless, the validation presented herein successfully captured the global strength and deformation capacity.

For each shear wall specimen, the load–displacement response predicted by the numerical model is plotted alongside the experimental curve, as shown in Figs. 30, 31, 32, 33, 34, 35, 36, 37, 38, 39, 40, 41, 42 allowing direct visual and quantitative assessment of the model’s performance. The degree of correlation is evaluated based on several metrics, including the closeness of initial stiffness, peak load prediction, post-peak descending branch, and displacement ductility.

**Fig. 30:**
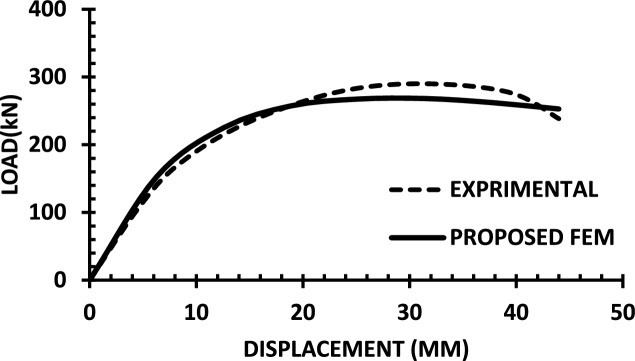
Wall 1 Experimental vs proposed FEM numerical results (SW2-Zhang et al.^22^).

**Fig. 31:**
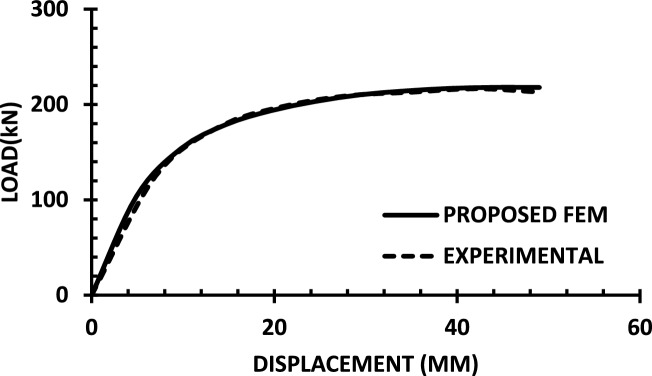
Wall 2 Experimental vs Proposed FEM numerical results (RH0.1F- Ni et al.^23^).

**Fig. 32:**
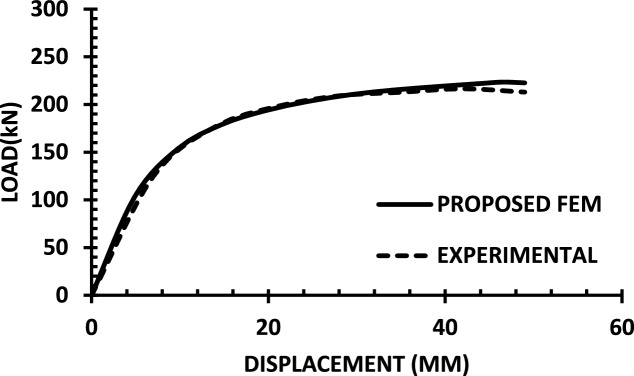
Wall 3 Experimental vs Proposed FEM numerical results (RH0.2F- Ni et al. ^23^.

**Fig. 33:**
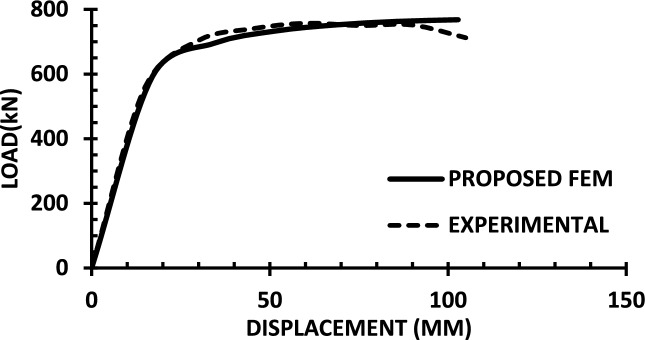
Wall 4 Experimental vs Proposed FEM numerical results (H 10-Bastami et al.^24^).

**Fig. 34:**
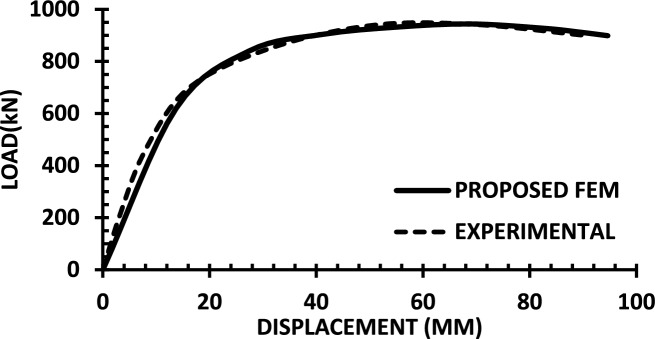
Wall 5 Experimental vs Proposed FEM numerical results (H 15-Bastami et al.^24^).

**Fig. 35:**
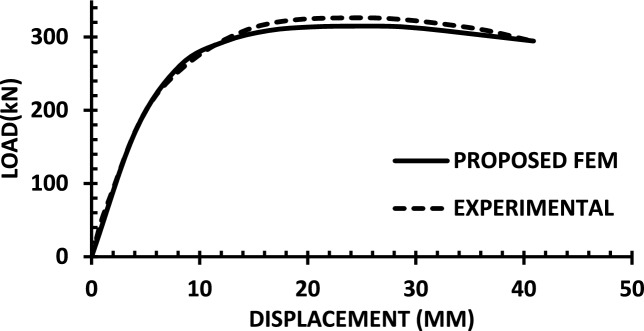
Wall 6 Experimental vs Proposed FEM numerical results (HPCW-01-Deng et al.^25^).

**Fig. 36:**
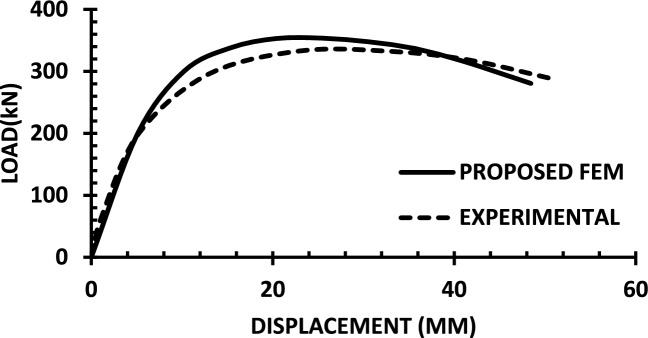
Wall 7 Experimental vs Proposed FEM numerical results (HPCW-02-Deng et al.^25^).

**Fig. 37:**
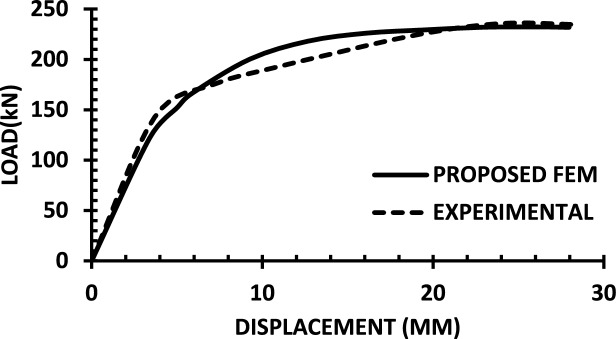
Wall 8 Experimental vs Proposed FEM numerical results (SW-2-Zhang et al.^26^).

**Fig. 38:**
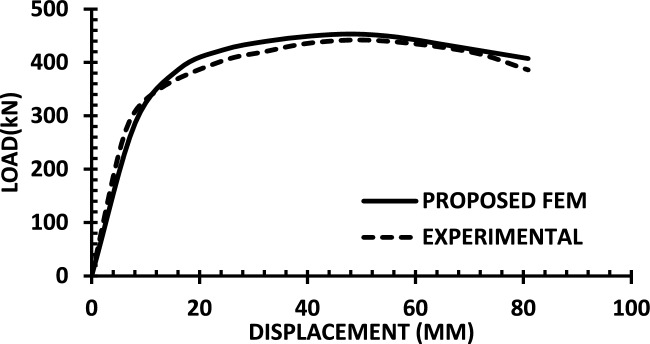
Wall 9 Experimental vs Proposed FEM numerical results (WR-20-Oh et al. ^27^).

**Fig. 39:**
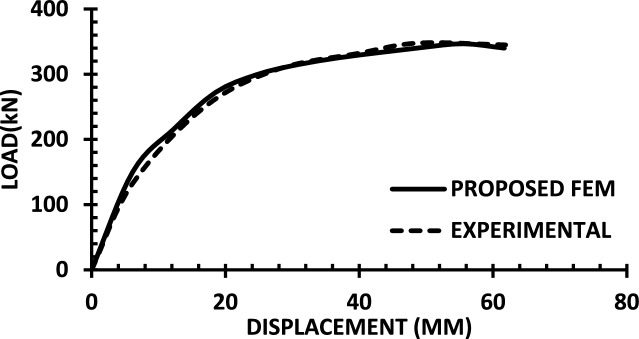
Wall 10 Experimental vs Proposed FEM numerical results (WSH2-Belmouden et al.^28^).

**Fig. 40:**
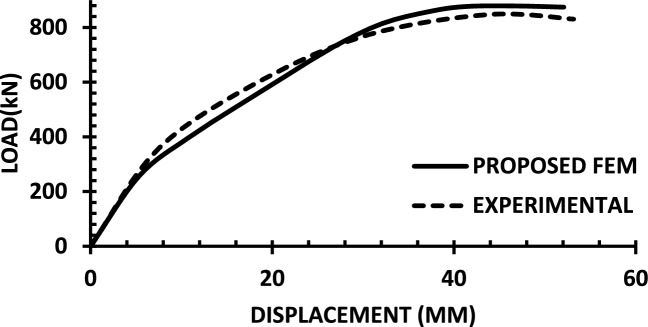
Wall 11 Experimental vs Proposed FEM numerical results (WSH2-Kim et al.^29^).

**Fig. 41:**
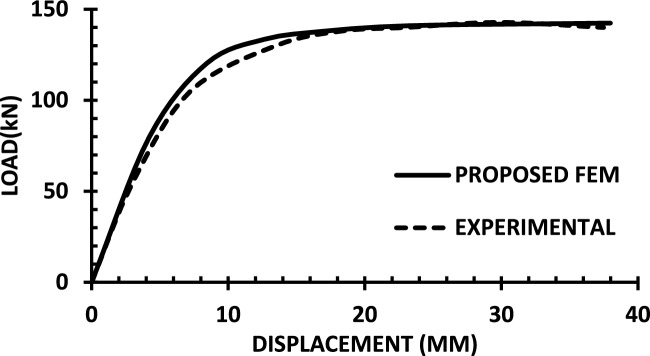
Wall 12 Experimental vs Proposed FEM numerical results (W1-Alarcon et al.^30^).

**Fig. 42:**
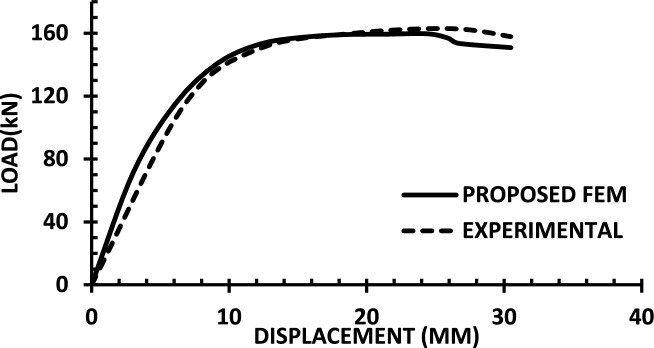
Wall 13 Experimental vs Proposed FEM numerical results(W2-Alarcon et al.^30^).

Table 10 presents a comparison between the experimental results and the proposed FEM method. The data demonstrate that the proposed FEM closely replicates the experimental behaviour across the full loading range, accurately capturing key response characteristics including initial stiffness, peak strength, and post-peak softening. This significant agreement highlights the model’s enhanced ability to simulate the complex nonlinear seismic response of reinforced concrete shear walls. In addition to its accuracy, the proposed FEM offers a simple and computationally efficient approach, overcoming the complexities and high computational demands of traditional analytical methods.

**Table 10:** Comparison between experimental and proposed FEM results.

Specimen		Cracking		Yielding		Peak		Ultimate	
		F (kN)	Δ (mm)	F (kN)	Δ (mm)	F (kN)	Δ (mm)	F (kN)	Δ (mm)
WALL 1	Experimental	127.5	5.4	200	17.8	301.8	33	255.1	46
	Proposed fem	137	5.5	237	14	270	31	238	44
	%Error	7%	2%	19%	− 21%	− 11%	− 6%	− 7%	− 4%
WALL 2	Experimental	90.55	5.71	179.52	16.76	218.13	43.95	213	50.66
	Numerical	99	6	185	16	220	43	217	51
	%Error	9%	5%	3%	− 5%	1%	− 2%	2%	1%
WALL 3	Experimental	141.13	6.21	197.3	12.77	256.6	38.38	254.92	40.32
	Proposed fem	138	5.8	193	11	238	30	234	40
	%Error	− 2%	− 7%	− 2%	− 14%	− 7%	− 22%	− 8%	− 1%
WALL 4	Experimental	594	17	695	34	751	90	767	103
	Numerical	560	15	704	30	762	85	712	105
	%Error	− 6%	− 12%	1%	− 12%	1%	− 6%	− 7%	2%
WALL 5	Experimental	702	16.4	858	32	941	69	898	91
	Proposed FEM	620	13.74	840	27.5	943	68	898	94
	%Error	− 12%	− 16%	− 2%	− 14%	0%	− 1%	0%	3%
WALL 6	Experimental	160	3.1	264	8.5	326	20	294	42
	Numerical	155	3.5	268	9	314	24	294	42
	%Error	− 3%	13%	2%	6%	− 4%	20%	0%	0%
WALL 7	Experimental	160	3.2	255	9	333	24	288	52
	Proposed FEM	144	3.3	295	10	317	30	278	50
	%Error	− 10%	3%	16%	11%	− 5%	25%	− 3%	− 4%
WALL 8	Experimental	142	3.7	175	7.2	236	24.6	200	35.5
	Numerical	122	3.4	200	9	232	27	226	35
	%Error	− 14%	− 8%	14%	25%	− 2%	10%	13%	− 1%
WALL 9	Experimental	273	6.3	348	12.4	441	53	386	80.9
	Proposed FEM	286	8	387	16	447	56	407	80.9
	%Error	5%	27%	11%	29%	1%	6%	5%	0%
WALL 10	Experimental	132	6	250	17	348	49	345	62
	Numerical	150	6.2	272	18.5	345	56	340	61.8
	%Error	14%	3%	9%	9%	− 1%	14%	− 1%	0%
WALL 11	Experimental	360	7.6	610	19	847	43.8	830	53
	Proposed FEM	332	8	594	20	878	46	871	54
	%Error	− 8%	5%	− 3%	5%	4%	5%	5%	2%
WALL 12	Experimental	105	7.2	130	13.5	141	29.5	142	38
	Numerical	120	8.4	134	12.66	142	29.9	140	37
	%Error	14%	17%	3%	− 6%	1%	1%	− 1%	− 3%
WALL 13	Experimental	62	3	115	6.8	163	26	157	30.5
	Proposed FEM	71	3.05	112	7	160	24.5	150	30
	%Error	15%	2%	− 3%	3%	− 2%	− 6%	− 4%	− 2%

A key advantage of the proposed FEM methodology is its capability to capture both the elastic response and the post-peak behaviour, which are critical for performance assessment. Initial stiffness governs the wall’s behaviour under service-level or moderate seismic loads, influencing building drift, vibration, and serviceability, while the post-peak response reflects progressive damage, energy dissipation, and ductility under strong seismic events, providing insight into ultimate capacity and structural resilience. By reliably reproducing these aspects, the proposed FEM serves as a practical and powerful tool for predicting full-range nonlinear behaviour, overcoming common modelling challenges, and providing engineers with practical solutions to simulate the actual behaviour of flexure-dominated RC walls.

## Conclusions

This study introduces an enhanced finite element modelling approach to accurately simulate the nonlinear flexural behaviour of reinforced concrete shear walls under seismic loading. The proposed model integrates fracture energy–based regularization with a four-line stiffness degradation framework, enabling a realistic representation of critical phenomena such as stiffness degradation, strain localization, and post-peak softening. These improvements effectively mitigate key limitations found in conventional Fiber-based modelling approaches, particularly issues related to mesh sensitivity and non-objective responses.

The proposed approach does not introduce a new finite element formulation but rather integrates fracture-energy regularization with a four-line stiffness degradation model to improve numerical objectivity and reduce mesh sensitivity.

Verification of the proposed model was conducted using data from thirteen reinforced concrete shear wall specimens tested in nine independent experimental programs. Despite employing monotonic pushover analysis in the numerical simulations, contrasting with the cyclic loading protocols of the experiments, the model demonstrated excellent agreement with observed load–displacement behaviour, including initial stiffness, peak strength, and post-peak response. This confirms the capability of pushover analysis with the proposed methodology to effectively capture essential seismic performance characteristics, providing a practical and computationally efficient alternative for performance-based seismic assessment.

By integrating the modified stress–strain relationship of concrete, including the adjusted Young’s modulus and fracture energy parameters, the proposed model provides a more realistic representation of material softening and failure progression. Consequently, the finite element model demonstrates improved agreement with experimental results and more accurately reproduces the overall pushover response of the tested walls.

Compared to traditional finite element methods the proposed framework offers significant improvements in both accuracy and reliability by directly incorporating refined constitutive material relationships for concrete and steel reinforcement. The differentiation between boundary and web reinforcement behaviours further enhances the model’s fidelity. As a result, this approach serves as a valuable tool for seismic design, retrofit, and evaluation of RC shear walls, bridging the gap between experimental observations and numerical predictions.

## Data Availability

All data generated or analysed during this study are included in this published article.
